# Multiparametric 0.4-T low-field MRI mapping for metabolic dysfunction-associated steatotic liver disease in rats

**DOI:** 10.1186/s41747-026-00852-6

**Published:** 2026-11-14

**Authors:** Kenichiro Okumura, Hibiki Yamauchi, Kazuto Kozaka, Naoki Ohno, Azusa Kitao, Taro Yamashita, Kenichi Harada, Yumie Takeshita, Toshinari Takamura, Satoshi Kobayashi

**Affiliations:** 1 Department of Radiology, Kanazawa University Graduate School of Medical Sciences, Kanazawa, Japan; 2 Faculty of Health Sciences, Institute of Medical, Pharmaceutical and Health Sciences, Kanazawa University, Kanazawa, Japan; 3 Department of Gastroenterology, Graduate School of Medical Sciences, Kanazawa, Japan; 4 Department of Human Pathology, Kanazawa University Graduate School of Medical Sciences, Kanazawa, Japan; 5 Department of Endocrinology and Metabolism, Kanazawa University Graduate School of Medical Sciences, Kanazawa, Japan

**Keywords:** Deep learning, Liver, Metabolic dysfunction-associated steatotic liver disease, Multiparametric magnetic resonance imaging, Rats (Wistar)

## Abstract

**Objective:**

Low-field MRI may broaden access to quantitative liver imaging, but low signal-to-noise ratio and coexisting steatosis, inflammation, fibrosis, and iron complicate relaxometry in metabolic dysfunction-associated steatotic liver disease (MASLD). We evaluated multiparametric 0.4-T MRI with deep-learning-reconstructed T1 mapping in rats.

**Materials and methods:**

In this prospective cross-sectional preclinical study, 90 male Wistar rats underwent 0.4-T liver MRI during dietary induction, progression, and recovery. Metrics included native T1, fat-corrected T1 (FcT1), T2, T2^*^, and an apparent proton density fat fraction (PDFF) estimate. Whole-section digital pathology quantified steatosis, fibrosis, inflammation, and iron. Associations were assessed using iron-adjusted and stage-aware analyses, multivariable regression with variance inflation factors, and receiver operating characteristic analysis.

**Results:**

Steatosis strongly correlated with T2 (ρ = 0.90, *p* < 0.001) and PDFF (ρ = 0.88, *p* < 0.001). For high inflammation, T2 and T2^*^ yielded areas under the curve (AUROCs) of 0.86 (95% confidence interval 0.78–0.94) and 0.80 (0.71–0.89), respectively (*p* = 0.294); specificities were 82.2% and 91.1%, respectively. For fibrosis, native T1 yielded higher AUROCs than FcT1 at collagen proportionate area (CPA) > 2.42% (0.77 *versus* 0.62; *p* = 0.008) and > 2.90% (0.78 *versus* 0.60; *p* = 0.001). Stage-aware analyses attenuated T1–fibrosis associations, and recovery animals showed residual fibrosis with near-baseline T1.

**Conclusion:**

Multiparametric 0.4-T MRI showed complementary, partly stage-dependent associations with MASLD pathology. T2/PDFF tracked steatosis; T2^*^ retained an inflammation-related association after stage adjustment; and T1 elevation characterized active high-burden progression rather than isolated residual fibrosis. Human validation and platform calibration are required.

**Key Points:**

***Question*** Noninvasive MASLD assessment needs accessible imaging biomarkers that account for coexisting steatosis, inflammation, fibrosis, and iron-related confounding in liver MRI.

***Findings*** Multiparametric 0.4-T MRI showed complementary but stage-dependent associations: T2/apparent PDFF tracked steatosis, T2^*^ related to inflammation, and T1 rose during high-burden progression.

***Relevance statement*** Multiparametric 0.4-T MRI provides complementary pathology-correlated measurements for MASLD assessment in rats, but reconstruction-dependent T1 bias and apparent PDFF estimation require platform calibration and human validation before clinical interpretation.

**Graphical Abstract:**

**Figure:**
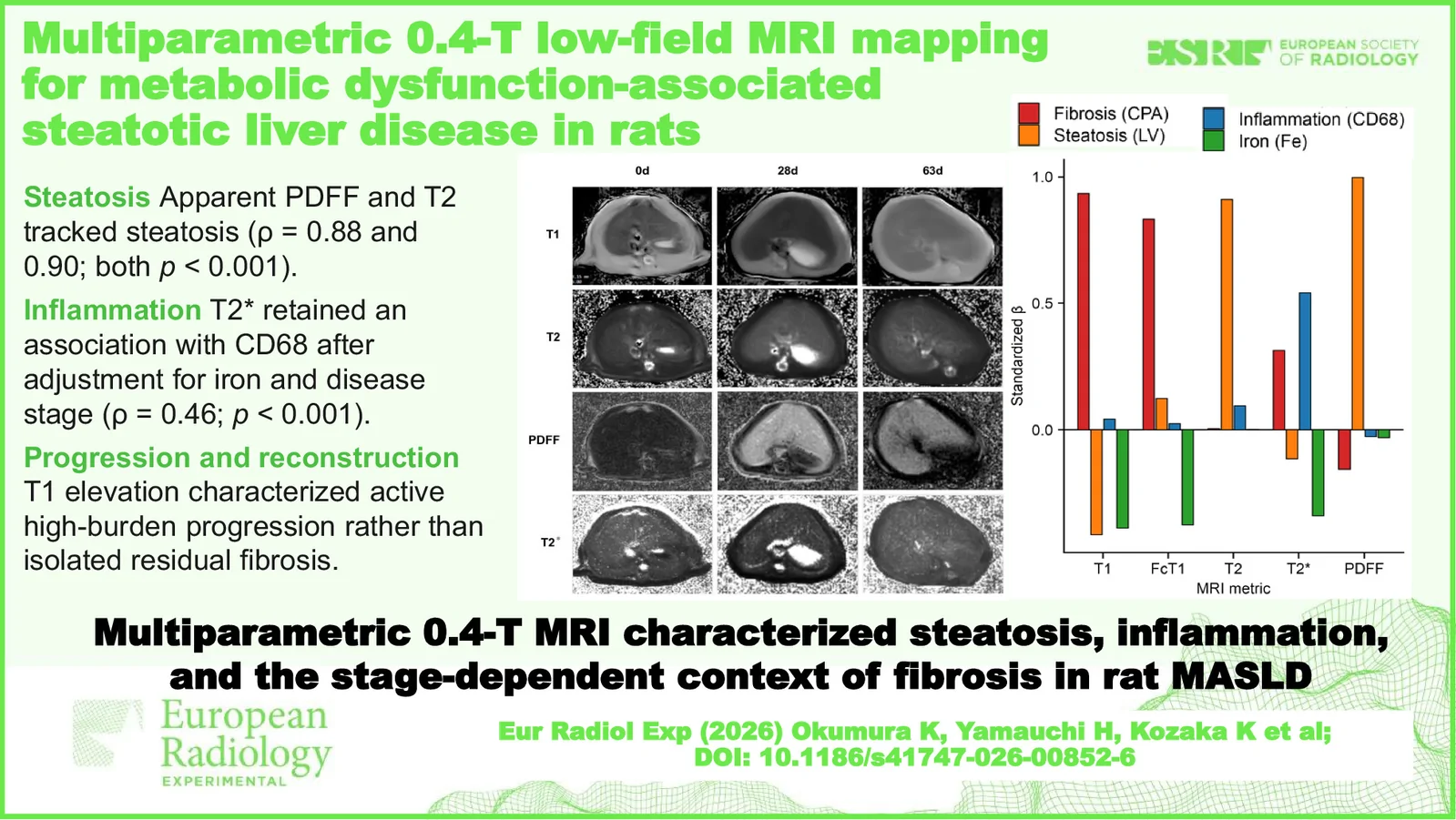

## Background

Metabolic dysfunction-associated steatotic liver disease (MASLD) requires non-invasive biomarkers that can characterize steatosis, inflammatory activity, and fibrosis while accounting for iron-related effects [1–8]. Proton density fat fraction (PDFF) is established for hepatic steatosis [9–14], although PDFF and histologic fat measurements can differ [15]. Liver relaxometry remains difficult because fat, extracellular matrix expansion, inflammatory water changes, and iron can alter T1, T2, and T2^*^ in different directions [16–23]. Therefore, individual magnetic resonance imaging (MRI) readouts may be sensitive to more than one tissue component rather than specific to a single pathological process.

Low-field MRI is a potential platform for accessible quantitative imaging because it can use less complex hardware, open-system designs, and lower infrastructure requirements [24, 25]. Field-strength effects on MR images have been described previously [26, 27]. We have therefore framed accessibility as a motivation and potential benefit rather than an outcome directly tested in this animal study. Low-field imaging has a lower signal-to-noise ratio. Because image-domain deep-learning reconstruction is nonlinear and may shift absolute values, its quantitative effects require direct validation and platform-specific calibration.

Dietary MASLD models can provide a broad preclinical pathology spectrum [28], and low-field hepatic iron assessment has been reported at 0.55 T [29]. Whole-section digital pathology is useful for preclinical MASLD imaging because it reduces sampling error compared with small biopsy-like samples [5, 30, 31]. We hypothesized that multiparametric 0.4-T MRI with deep-learning-reconstructed T1 mapping would provide complementary, pathology-correlated readouts in a rat MASLD model. The purpose of this prospective preclinical study was to evaluate native T1, fat-corrected T1 (FcT1), T2, T2^*^, and PDFF against whole-section digital pathology during dietary induction, progression, and recovery, while explicitly assessing stage-related confounding and collinearity.

## Materials and methods

### Study design and animal model

This prospective cross-sectional preclinical study complied with ARRIVE guidelines and was approved by the institutional animal ethics committee (approval number AP23009). Healthy male Wistar rats were assigned to prespecified terminal endpoints without randomization. MASLD was induced using a choline-deficient, L-amino acid-defined, high-fat diet–CDAHFD. The analysis set comprised 90 rats after excluding 2 animals that died before MRI and tissue collection and 13 prespecified reserves that were not needed. Groups included acute induction (day 0, 2, and 4; *n* = 6 each), progression (day 7, 14, 28, and 63; *n* = 12 each), and dietary recovery (F4N20 and F9N15; *n* = 12 each). F4N20 and F9N15 indicated choline-deficient, L-amino acid-defined, high-fat diet–CDAHFD feeding for 4 or 9 weeks followed by normal chow for 20 or 15 weeks, respectively.

### MRI acquisition and reconstruction

MRI was performed under intraperitoneal medetomidine/midazolam/butorphanol anesthesia on a clinical 0.4-T open MRI system (Gravity MRI, FUJIFILM Healthcare). Quantitative metrics were native T1, FcT1, T2, T2^*^, and an apparent PDFF estimate. T1/FcT1 mapping combined a two-dimensional inversion-recovery fast spin-echo acquisition (repetition time 2,000 ms; inversion time 600 ms; echo time 15.0 ms) with the first-echo image of the two-echo fast spin-echo acquisition (echo time 15.0 ms), which served as the inversion time 0-ms reference. T2 mapping used a two-echo fast spin-echo acquisition with echo times of 15 and 60 ms and monoexponential apparent T2 estimation. At 0.4 T, this apparent T2 should be interpreted as a sequence-specific composite measure because no water/fat separation or fat suppression was applied and two echoes do not permit robust voxel-wise residual or goodness-of-fit assessment. T2^*^ and the apparent PDFF estimate were derived from a low-flip-angle multiecho gradient-echo acquisition (repetition time 250 ms; echo times 8.9, 17.8, 26.7, and 35.6 ms; flip angle 12°) using a simplified two-component magnitude water–fat signal model with a fixed nominal chemical-shift term. No voxel-wise B_0_ field map or off-resonance correction was acquired or applied. Accordingly, PDFF herein denotes an apparent estimate. Additional acquisition parameters and equations are provided in Supplementary Methods.

Image-domain deep-learning reconstruction (SwiftMR version 3.0.13.1; AIRS Medical) was applied only to T1 mapping because the low-field two-point inversion-recovery acquisition had the lowest signal-to-noise ratio, and this was the available reconstruction workflow. T2, T2^*^, and PDFF used the system’s standard reconstruction. FcT1 was derived from the two-point inversion-recovery data using a PDFF-informed exponential mixing model with fixed fat T1 of 200 ms. Technical validation of T1 mapping, including phantom cross-calibration, AIRS ON–OFF effects, flip-angle sensitivity, and normal-rat repeatability, is summarized in Supplementary Appendix P.

### Image analysis and pathology

Regions of interest were drawn on a central liver slice while avoiding large vessels, gallbladder, and visible artifacts. Three circular liver regions of interest were propagated across maps when possible, and the mean value was used. Although tissue blocks were sampled from liver regions corresponding as closely as feasible to the central MRI slice, exact voxel-level MRI–histology co-registration was not possible; this spatial mismatch was considered when interpreting correlations.

Whole-section liver histology was performed after euthanasia. Hematoxylin and eosin sections quantified steatosis as lipid vacuole area fraction (LV). Sirius Red quantified fibrosis as collagen proportionate area (CPA). CD68 immunohistochemistry quantified macrophage-rich inflammation, and Perls’ Prussian blue quantified Fe (Perls’ Prussian blue-positive area fraction). Whole-slide images were digitized and analyzed in QuPath version 0.5.1 using stain-specific thresholds. MRI and pathology analyses were performed by separate investigators blinded to the other modality.

### Diagnostic targets and statistical analysis

High inflammation was defined as CD68-positive area fraction (CD68) > 2.33%, the cohort median, to provide a balanced within-cohort receiver operating characteristic target. Fibrosis operating points were defined as CPA > 1.95%, > 2.42%, and > 2.90% by shifting published human non-alcoholic fatty liver disease CPA distributions/cutoffs by the day 0 median CPA [32]. These thresholds were used only as within-cohort operating points and not as species conversions or clinical cutoffs.

Continuous variables are presented as mean (standard deviation). Between-group differences were assessed using Kruskal–Wallis testing followed by Dunn’s test with Benjamini–Hochberg adjustment. Associations between MRI and pathology metrics were evaluated using Spearman partial correlations adjusted for Fe. Standardized multivariable linear regression modeled each MRI metric using Fe, LV, CPA, and CD68 as simultaneous predictors. Variance inflation factors (VIFs) were calculated to assess collinearity among pathology predictors. Because pathological components co-evolved with experimental stage, additional conservative sensitivity analyses included timepoint/group as categorical covariates. Receiver operating characteristic analysis assessed discrimination of high inflammation and fibrosis thresholds; area under the receiver operating characteristic curve (AUROCs) were estimated and compared using paired DeLong tests. The AIRS technical analyses were exploratory and descriptive; no formal superiority or inferiority test was prespecified. Rank ordering of standardized regression coefficients was also descriptive, and no pairwise tests between coefficients were performed. Exact *p*-values are reported to three decimals where applicable; values smaller than 0.001 are reported as *p* < 0.001.

### Use of large language models

ChatGPT (OpenAI) was used after data analysis to assist with English editing, manuscript-formatting checks, and drafting response-letter text. It was not used to generate, analyze, or interpret research data, statistical output, images, or videos. All artificial intelligence-assisted text was critically reviewed and edited by the authors.

## Results

### Disease trajectories during induction and recovery

**Fig. 1:**
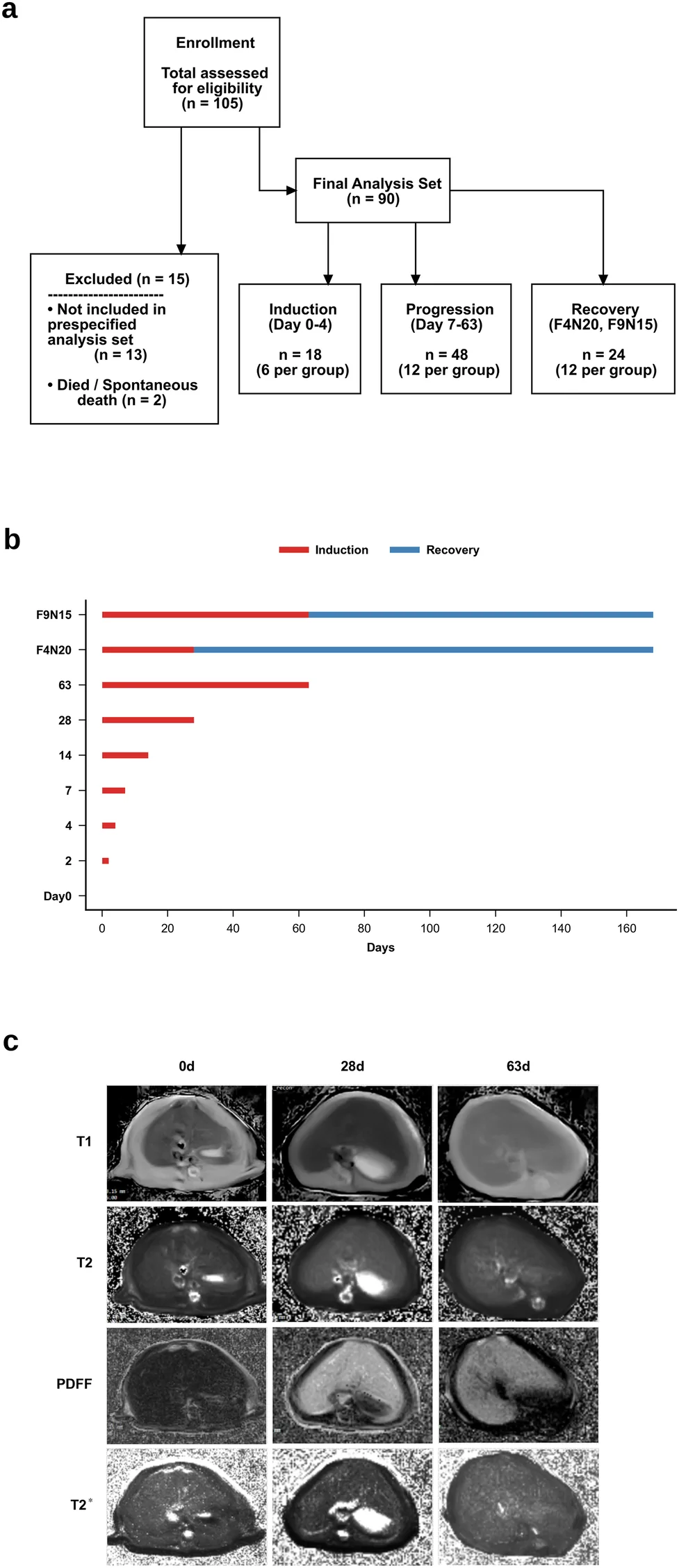
Study design and representative MRI appearances in a MASLD rat model. **a** Of 105 enrolled rats, 90 were analyzed; 13 reserves were unused, and 2 day 63 rats died before MRI and tissue sampling. **b** Animals were allocated to terminal endpoint groups at day 0, 2, 4, 7, 14, 28, 63, F4N20, or F9N15. **c** Representative axial T1, T2, T2^*^, and PDFF maps are shown for days 0, 28, and 63. F4N20 and F9N15 indicated choline-deficient, L-amino acid-defined, high-fat diet–CDAHFD feeding for 4 or 9 weeks followed by normal chow for 20 or 15 weeks, respectively. MASLD, Metabolic dysfunction-associated steatotic liver disease; MRI, Magnetic resonance imaging; PDFF, Proton density fat fraction (apparent estimate)

**Fig. 2:**
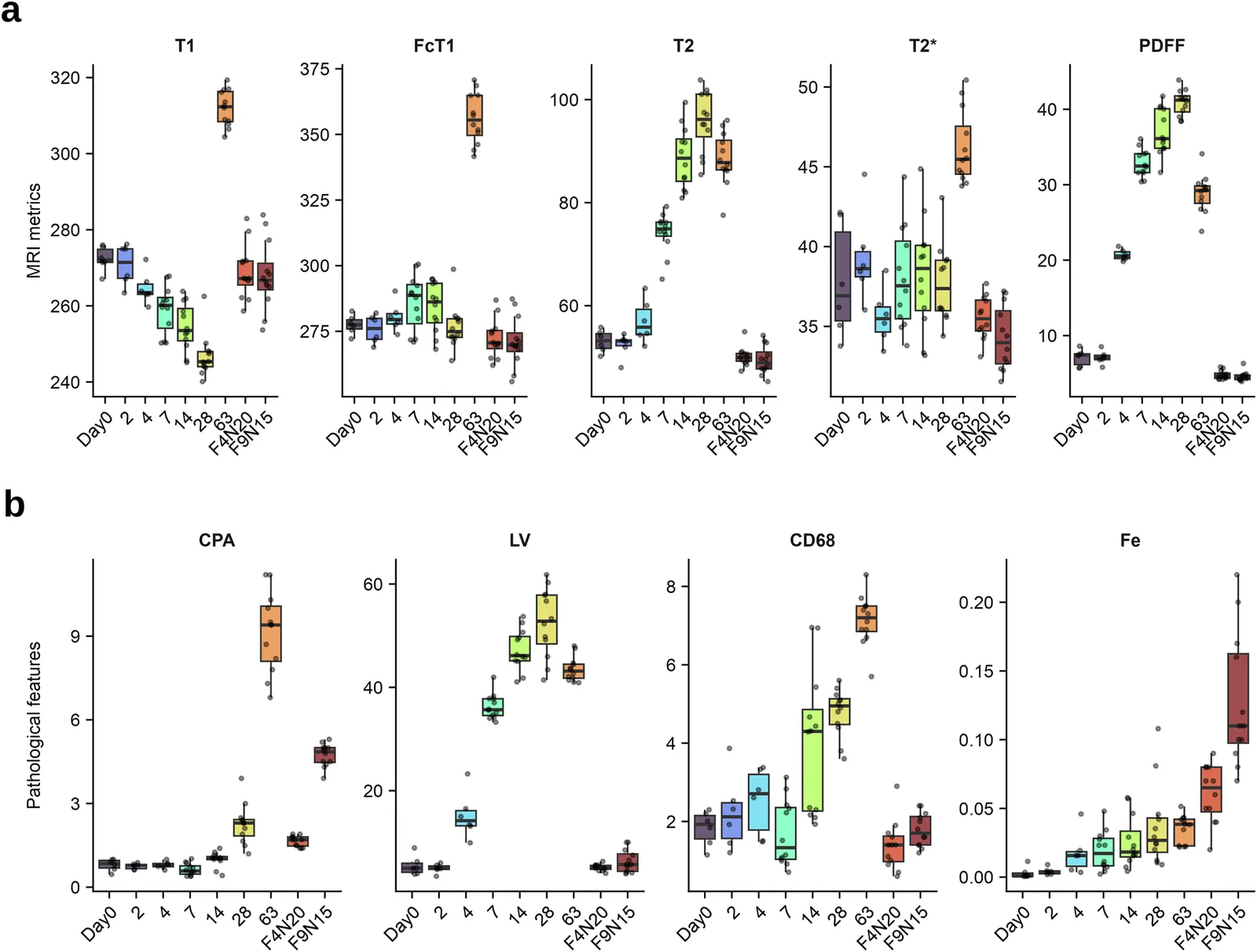
Changes in MRI and histologic metrics across experimental groups. **a** Box plots show T1, FcT1, T2, T2^*^, and PDFF. **b** Corresponding histologic metrics are LV, CPA, CD68, and Fe-positive area fraction. Points indicate individual rats; boxes, median and interquartile range; whiskers, 1.5 × interquartile range. Group differences were tested with Kruskal–Wallis and Dunn’s test with Benjamini–Hochberg correction. F4N20 and F9N15 indicated choline-deficient, L-amino acid-defined, high-fat diet–CDAHFD feeding for 4 or 9 weeks followed by normal chow for 20 or 15 weeks, respectively. CPA, Collagen proportionate area; FcT1, Fat-corrected T1; LV, Lipid vacuole area fraction; MRI, Magnetic resonance imaging; PDFF, Proton density fat fraction (apparent estimate)

The final cohort included 90 rats: 18 acute-induction, 48 progression, and 24 recovery animals (Fig. 1). Quantitative MRI and histologic metrics changed across experimental groups (all global *p* < 0.001;  Fig. 2; Tables 1 and 2). PDFF increased from 7.1 (1.1)% at day 0 to 40.9 (1.7)% at day 28 and declined to 28.8 (2.5)% at day 63. Histologic steatosis showed a parallel trajectory, increasing from 5.5 (1.9)% to 52.5 (6.7)% at day 28. Fibrosis and inflammation increased later, with CPA reaching 9.2 (1.4)% and CD68 reaching 7.1 (0.7)% at day 63. During recovery, PDFF and LV returned near baseline, whereas fibrosis persisted, especially in F9N15 (CPA, 4.7 [0.4]%).

**Table 1:** Quantitative MRI metrics across experimental time points and recovery groups

Group	T1 (ms)	FcT1 (ms)	T2 (ms)	T2^*^ (ms)	PDFF (%)
Day 0	272.4 (3.3)	277.5 (3.6)	53.1 (2.1)	37.8 (3.5)	7.1 (1.1)
Day 2	270.7 (5.3)	275.8 (5.3)	52.4 (2.3)	39.3 (2.9)	7.1 (0.9)
Day 4	264.7 (4.2)	280.5 (5.6)	56.9 (4.2)	35.6 (1.7)	20.7 (0.7)
Day 7	259.0 (6.2)^*^	286.0 (10.5)	74.1 (3.9)	38.0 (3.2)	32.9 (1.8)^*^
Day 14	254.5 (6.2)^**^	284.3 (9.4)	88.7 (6.0)^**^	38.3 (3.5)	37.0 (3.1)^**^
Day 28	246.8 (5.6)^***^	276.7 (8.4)	95.7 (5.9)^***^	37.7 (2.4)	40.9 (1.7)^***^
Day 63	312.0 (4.8)	356.0 (9.7)^**^	88.5 (5.1)^**^	46.2 (2.3)^**^	28.8 (2.5)
F4N20	269.0 (7.1)	272.2 (7.4)	50.1 (1.9)	35.5 (1.3)	4.8 (0.6)
F9N15	268.0 (9.2)	271.0 (9.6)	49.4 (2.8)	34.3 (2.0)	4.6 (0.6)
*p-*value	< 0.001	< 0.001	< 0.001	< 0.001	< 0.001

Values are mean (standard deviation); *p*-values represent global Kruskal–Wallis tests. Asterisks indicate significant differences *versus* day 0 after Dunn’s test with Benjamini–Hochberg adjustment: ^*^ *p* < 0.05, ^**^ *p* < 0.01, ^***^ *p* < 0.001. F4N20 and F9N15 indicated choline-deficient, L-amino acid-defined, high-fat diet–CDAHFD feeding for 4 or 9 weeks followed by normal chow for 20 or 15 weeks, respectively

*FcT1* Fat-corrected T1, *PDFF* Proton density fat fraction (apparent estimate)

**Table 2:** Quantitative histopathology metrics across experimental timepoints and recovery groups

Group	Steatosis (LV %)	Fibrosis (CPA %)	Inflammation (CD68%)	Iron (Fe %)
Day 0	5.5 (1.9)	0.8 (0.2)	1.8 (0.4)	0.00 (0.00)
Day 2	4.9 (0.9)	0.8 (0.1)	2.2 (1.0)	0.00 (0.00)
Day 4	15.2 (4.5)	0.8 (0.1)	2.5 (0.8)	0.02 (0.02)
Day 7	36.3 (2.4)^*^	0.6 (0.2)	1.7 (0.8)	0.02 (0.01)
Day 14	47.2 (4.0)^***^	1.0 (0.3)	4.0 (1.8)^*^	0.03 (0.02)^*^
Day 28	52.5 (6.7)^***^	2.3 (0.7)^**^	4.8 (0.6)^**^	0.04 (0.03)^**^
Day 63	43.5 (2.4)^***^	9.2 (1.4)^***^	7.1 (0.7)^***^	0.04 (0.01)^**^
F4N20	5.1 (0.6)	1.6 (0.2)^*^	1.4 (0.6)	0.06 (0.02)^***^
F9N15	6.3 (2.2)	4.7 (0.4)^***^	1.8 (0.4)	0.13 (0.05)^***^
*p*-value	< 0.001	< 0.001	< 0.001	< 0.001

Values are means (standard deviation); *p*-values represent global Kruskal–Wallis tests. Asterisks indicate significant differences *versus* day 0 after Dunn’s test with Benjamini–Hochberg adjustment: ^*^ *p* < 0.05, ^**^ *p* < 0.01, ^***^ *p* < 0.001. F4N20 and F9N15 indicated choline-deficient, L-amino acid-defined, high-fat diet–CDAHFD feeding for 4 or 9 weeks followed by normal chow for 20 or 15 weeks, respectively

*CPA* Collagen proportionate area, *Fe* Perls’ Prussian blue-positive area fraction, *LV* Lipid vacuole area fraction

Native T1 decreased during steatosis-dominant disease (272.4 [3.3] ms at day 0 to 246.8 [5.6] ms at day 28) and then increased markedly by day 63 (312.0 [4.8] ms). FcT1 increased to 356.0 (9.7) ms at day 63. During recovery, T1 returned near baseline despite residual fibrosis in F9N15 (268.0 [9.2] ms; CPA, 4.7 [0.4]%), indicating that T1 elevation was not a simple marker of residual collagen burden. Region-of-interest reproducibility was excellent, with intraobserver intraclass correlation coefficients of 0.99–1.00 and interobserver coefficients of 0.95–1.00.

### Iron-adjusted and stage-aware associations

**Fig. 3:**
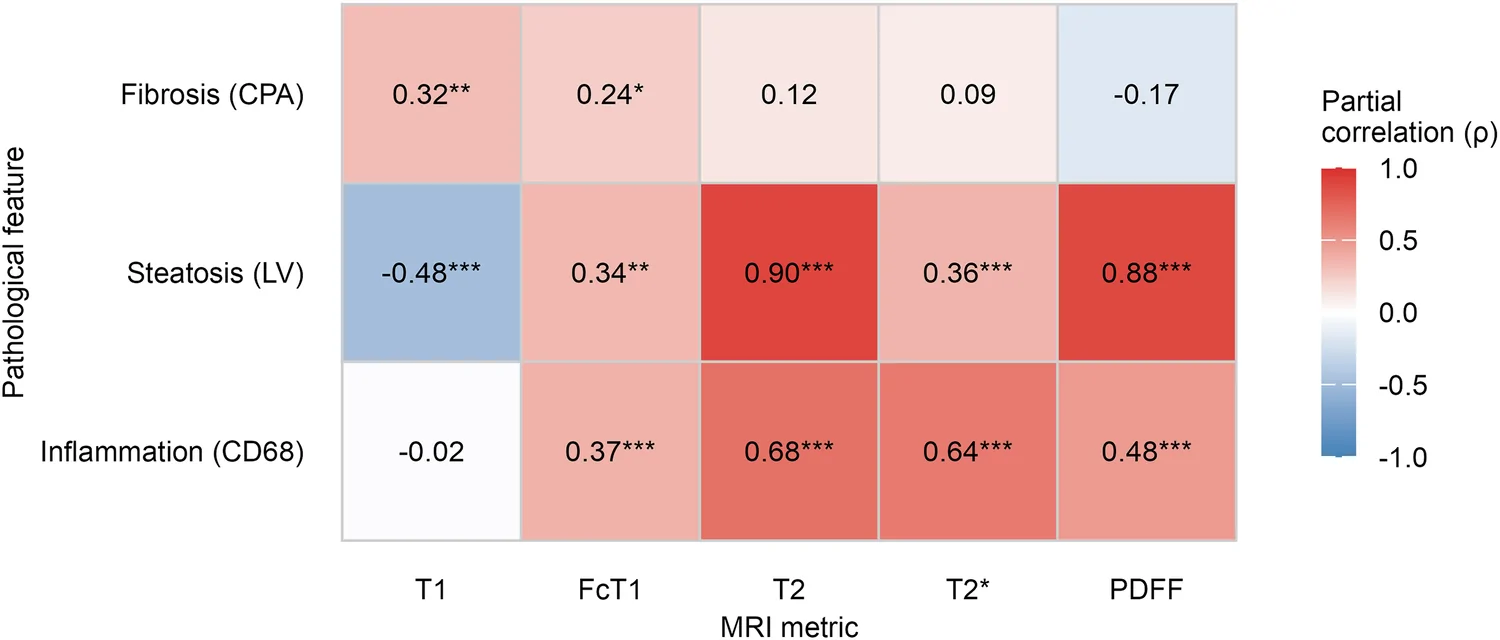
Iron-adjusted partial correlations between MRI and pathology metrics. The heat map shows Spearman partial correlation coefficients between T1, FcT1, T2, T2^*^, and PDFF and the pathology metrics LV, CPA, and CD68, with Fe included as a covariate but not displayed. These are Fe-adjusted analyses, not stage- or co-pathology-adjusted analyses. Cell color indicates direction and magnitude; values are rho. Asterisks indicate significance from partial-correlation tests: ^*^p < 0.05, ^**^p < 0.01, ^***^p < 0.001. CPA, Collagen proportionate area; FcT1, Fat-corrected T1; LV, Lipid vacuole area fraction; MRI, Magnetic resonance imaging; PDFF, Proton density fat fraction (apparent estimate)

After adjustment for timepoint/group, Fe added negligible incremental variance to MRI metrics (Δ*R*² ≤ 0.001; all false-discovery-rate-adjusted *q* > 0.05). In Fe-adjusted partial correlations (Fig. 3), LV correlated strongly with T2 (ρ = 0.90, *p* < 0.001) and PDFF (ρ = 0.88, *p* < 0.001). CD68 correlated with T2 (ρ = 0.68, *p* < 0.001) and T2^*^ (ρ = 0.64, *p* < 0.001). CPA correlated modestly with T1 (ρ = 0.32, *p* = 0.002) and FcT1 (ρ = 0.24, *p* = 0.020).

When analyses were adjusted for timepoint/group, the associations changed substantially. T2 remained associated with steatosis (ρ = 0.34, *p* = 0.002), whereas its CD68 association attenuated (ρ = 0.07, *p* = 0.556). T2^*^ retained an inflammation-related association (ρ = 0.46, *p* < 0.001). T1 and FcT1 associations with CPA were no longer evident after timepoint/group adjustment, supporting a stage-dependent interpretation of T1-based fibrosis discrimination.

**Fig. 4:**
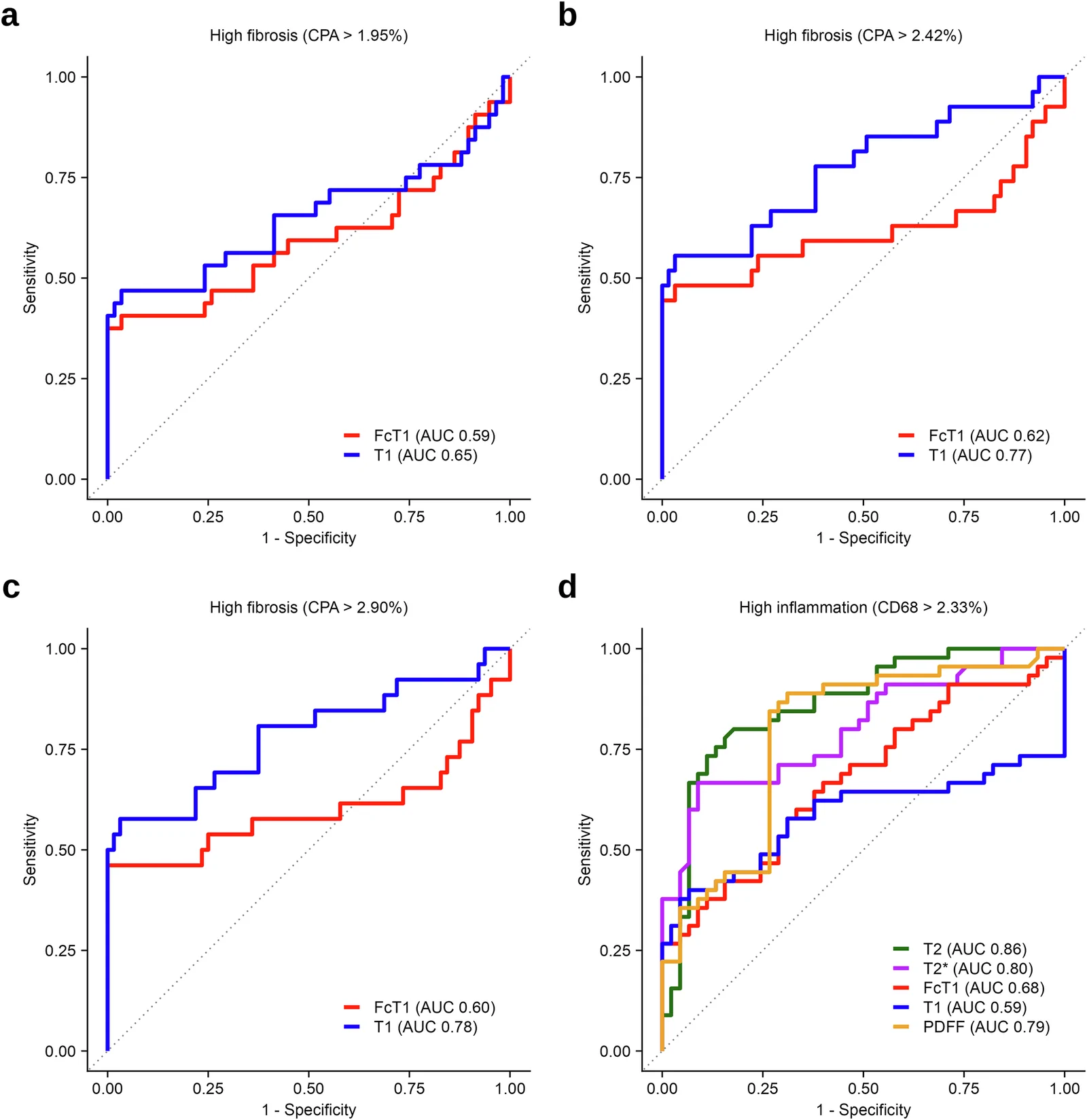
Receiver operating characteristic performance for fibrosis and high inflammation. **a**–**c** Fibrosis was defined as CPA > 1.95%, > 2.42%, and > 2.90%. **d** High inflammation was CD68 > 2.33%. AUROCs with 95% confidence intervals were calculated using the DeLong method, optimal cutoffs by the Youden index, and paired AUROCs compared with the DeLong test. AUC: Area under the receiver operating characteristic curve; CPA, Collagen proportionate area; FcT1, Fat-corrected T1; PDFF, Proton density fat fraction (apparent estimate)

### Discrimination of high inflammation and fibrosis

For high inflammation (CD68 > 2.33%), T2 achieved an AUROC of 0.86 (95% confidence interval 0.78–0.94) at a 74.1-ms cutoff, with sensitivity of 80.0% (36/45) and specificity of 82.2% (37/45). T2^*^ achieved an AUROC of 0.80 (0.71–0.89), with sensitivity of 66.7% (30/45) and specificity of 91.1% (41/45) (Fig. 4d; Table 3). The AUROC difference was not statistically significant (*p* = 0.294), and sensitivity and specificity were not formally compared between metrics.

**Table 3:** Diagnostic performance of quantitative MRI metrics for high inflammation and fibrosis

Target	Metric	AUROC (95% CI)	Cutoff	Sensitivity	Specificity	Comparison
High inflammation (CD68 > 2.33%)	T1	0.59 (0.46–0.71)	253.8	18/45 (40.0% [27.0–54.5])	42/45 (93.3% [82.1–97.7])	—
	T2	0.86 (0.78–0.94)	74.1	36/45 (80.0% [66.2–89.1])	37/45 (82.2% [68.7–90.7])	0.294 (*vs* T2^*^)
	T2^*^	0.80 (0.71–0.89)	38.5	30/45 (66.7% [52.1–78.6])	41/45 (91.1% [79.3–96.5])	—
	FcT1	0.68 (0.57–0.79)	278.6	30/45 (66.7% [52.1–78.6])	27/45 (60.0% [45.5–73.0])	—
	PDFF	0.79 (0.69–0.88)	20.3	39/45 (86.7% [73.8–93.7])	32/45 (71.1% [56.6–82.3])	—
Fibrosis (CPA > 1.95%)	T1	0.65 (0.51–0.79)	276.8	15/32 (46.9% [30.9–63.6])	56/58 (96.6% [88.3–99.0])	0.381 (*vs* FcT1)
	FcT1	0.59 (0.45–0.74)	321.1	12/32 (37.5% [22.9–54.7])	58/58 (100.0% [93.8–100.0])	—
Fibrosis (CPA > 2.42%)	T1	0.77 (0.65–0.89)	276.8	15/27 (55.6% [37.3–72.4])	61/63 (96.8% [89.1–99.1])	0.008 (*vs* FcT1)
	FcT1	0.62 (0.45–0.78)	296.8	13/27 (48.1% [30.7–66.0])	61/63 (96.8% [89.1–99.1])	—
Fibrosis (CPA > 2.90%)	T1	0.78 (0.66–0.91)	276.8	15/26 (57.7% [38.9–74.5])	62/64 (96.9% [89.3–99.1])	0.001 (*vs* FcT1)
	FcT1	0.60 (0.43–0.77)	321.1	12/26 (46.2% [28.8–64.5])	64/64 (100.0% [94.3–100.0])	—

High inflammation was defined as CD68 > 2.33%. Fibrosis was evaluated at CPA > 1.95%, > 2.42%, and > 2.90%. AUROCs with 95% CI were calculated using the DeLong method; optimal cutoffs were determined using the Youden index. Sensitivity and specificity are shown as numerator/denominator with percentages and 95% Wilson CI. AUROC, Area under the receiver operating characteristic curve; CI, confidence interval; CPA, collagen proportionate area; FcT1, fat-corrected T1; PDFF, proton density fat fraction (apparent estimate)

For fibrosis, native T1 yielded higher AUROCs than FcT1 at stricter CPA thresholds. At CPA > 2.42%, AUROCs were 0.77 for T1 and 0.62 for FcT1 (*p* = 0.008). At CPA > 2.90%, AUROCs were 0.78 and 0.60, respectively (*p* = 0.001). FcT1 maintained high specificity, ranging from 96.8% to 100.0% across thresholds (Fig. 4a–c; Table 3). Because sensitivity was limited and recovery fibrosis was not consistently detected by T1, these results were interpreted as within-cohort discrimination of the active high-burden progression endpoint rather than as validated fibrosis cutoffs.

### Multivariable models and collinearity

**Fig. 5:**
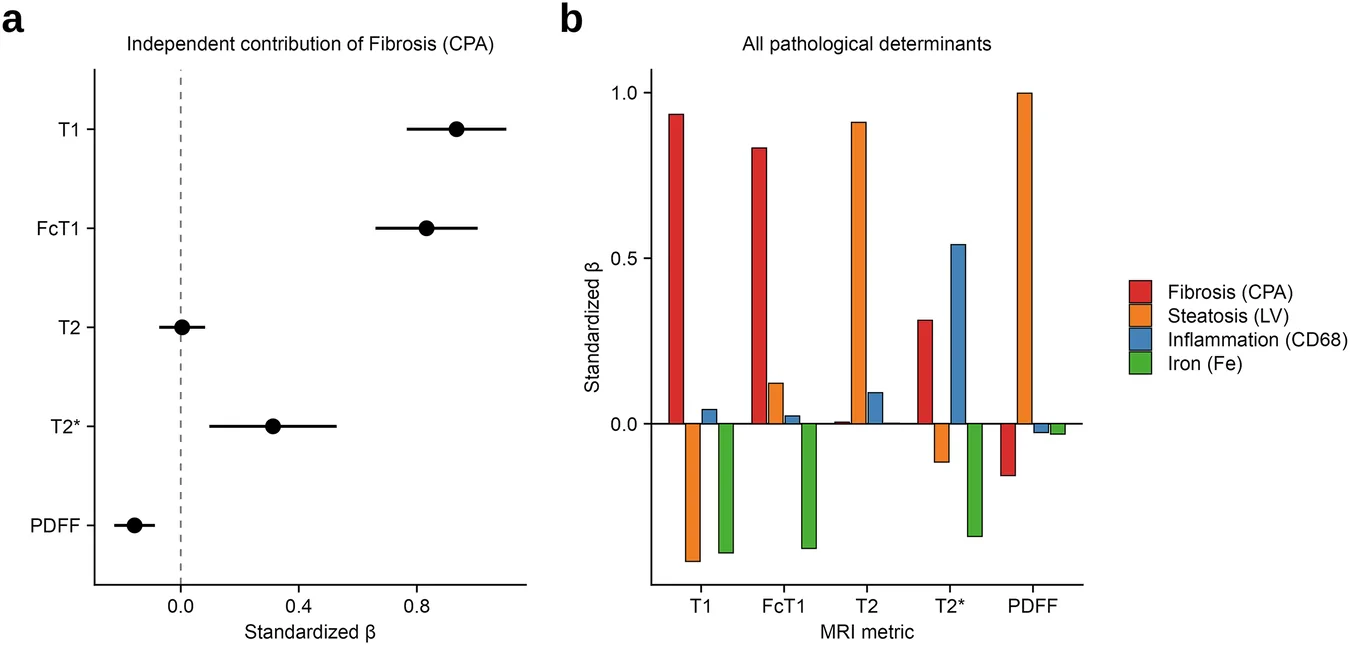
Pathological determinants of MRI metrics from standardized multivariable regression. **a** Forest plot shows the association of CPA with each MRI metric after adjustment for Fe, LV, and CD68. **b** Bar plot shows standardized coefficients for Fe, CPA, LV, and CD68 in models of T1, FcT1, T2, T2^*^, and PDFF. Error bars in panel a denote 95% confidence intervals. Detailed beta, 95% confidence intervals, and *p*-values are provided in Supplementary Table S2. Pathology-only VIFs were Fe 1.53, LV 2.05, CPA 2.61, and CD68 3.74; timepoint-adjusted models showed higher VIFs and were interpreted as sensitivity analyses. CPA, Collagen proportionate area; FcT1, Fat-corrected T1; Fe, Perls’ Prussian blue-positive area fraction; LV, Lipid vacuole area fraction; MRI, Magnetic resonance imaging; PDFF, Proton density fat fraction (apparent estimate); VIF, Variance inflation factor

In standardized multivariable regression including Fe, LV, CPA, and CD68, the observed standardized coefficients were 0.93 for CPA with T1, 0.83 for CPA with FcT1, 0.91 for LV with T2, 1.00 for LV with PDFF, and 0.54 for CD68 with T2^*^. CPA and Fe also contributed to T2^*^ (Fig. 5). These coefficient rankings were descriptive and were not compared using pairwise inferential tests.

VIFs for the pathology-only models were 1.53 for Fe, 2.05 for LV, 2.61 for CPA, and 3.74 for CD68, indicating acceptable collinearity for Fig. 5b. In timepoint/group-adjusted sensitivity models, pathology-predictor VIFs increased to 3.72 for Fe, 37.19 for LV, 23.58 for CPA, and 5.99 for CD68, reflecting strong co-evolution of pathology with disease stage. Therefore, timepoint-adjusted coefficients were interpreted as conservative sensitivity analyses rather than stable estimates of pathology-specific effects.

## Discussion

This preclinical study paired whole-section digital pathology with multiparametric 0.4-T MRI to evaluate how low-field MRI metrics relate to steatosis, inflammation, fibrosis, and iron in a rat MASLD model. Although T1 and T2 mapping have been studied as fibrosis-related techniques in preclinical liver models [33], the revised interpretation is deliberately cautious. Rather than showing pathology-specific biomarkers, the data show complementary but partly stage-dependent pathology-correlated readouts. T2 and PDFF primarily tracked steatosis, T2^*^ retained an inflammation-related association after stage-aware analyses, and T1 elevation characterized the active high-burden progression state rather than isolated residual fibrosis.

The role of T2 requires caution because macrophage-rich inflammation and portal inflammatory changes are relevant to MASH pathology [34, 35]. Although T2 had a numerically higher AUROC than T2^*^ for the cohort-defined high-inflammation endpoint, the difference was not statistically significant (*p* = 0.294), and multivariable and stage-aware analyses indicated that T2 variance was largely driven by steatosis. This is plausible because T2 mapping was performed without water/fat separation or fat suppression, and fat accumulation can influence apparent T2. Therefore, T2 should be interpreted as a sensitive marker of the steatosis-rich inflammatory progression state in this model, not as a specific inflammation marker. At the selected cutoffs, sensitivity and specificity were 80.0% and 82.2% for T2 and 66.7% and 91.1% for T2^*^, respectively; these proportions were not formally compared. T2^*^ retained a CD68 association after timepoint/group adjustment, supporting an inflammation-related role. The counterintuitive increase in T2^*^ at day 63 may reflect inflammation-related tissue water and architectural changes dominating over the relatively small iron burden observed in this model; direct multi-field and iron-overload validation remains necessary.

The recovery groups provided an important stress test for fibrosis interpretation. In F9N15 animals, CPA remained elevated despite near-normalization of PDFF, LV, T2, T2^*^, and native T1. This indicates that native T1 elevation was not a simple marker of residual collagen burden. Instead, T1 elevation appeared most closely linked to the active day 63 high-burden endpoint, where fibrosis, inflammation, tissue-water changes, and disease stage co-occurred. The higher T1 AUROCs at stricter CPA thresholds are therefore best understood as within-cohort discrimination of active high-burden progression, not as evidence of a fibrosis-specific or clinically transferable cutoff. FcT1 retained very high specificity, suggesting a possible rule-in role, but its sensitivity was limited in this model. High-resolution structural imaging addresses a different aspect of fibrosis: Dana et al used *ex vivo* 7-T fat-suppressed T2-weighted MRI to visualize liver microarchitecture and stage fibrosis against histopathology [36]. This differs from our *in vivo* 0.4-T relaxation measurements, which did not directly resolve fibrotic architecture.

Deep-learning reconstruction was used as the available workflow for the low-signal-to-noise two-point inversion-recovery T1 acquisition, not as a demonstrated quantitative improvement. In normal-rat liver, AIRS ON produced a mean 2ptIR T1 increase of 10.64 ms relative to AIRS OFF (95% limits of agreement, -0.31 to 21.58 ms). Test–retest descriptors were numerically less favorable with AIRS ON than OFF (repeatability coefficient, 15.28 *versus* 5.71 ms; within-subject coefficient of variation, 1.99% *versus* 0.77%; intraclass correlation coefficient, 0.15 *versus* 0.84), although no formal superiority or inferiority test was prespecified. Because native T1 differences across disease stages can be only several to several tens of milliseconds, this reconstruction-dependent shift may affect absolute-value interpretation and threshold transfer. These exploratory data characterize AIRS-related measurement effects but do not establish the usefulness of AIRS for quantitative T1 mapping. Platform-specific calibration is therefore required. Automation of measurement is a separate consideration. Zbinden et al combined deep-learning segmentation with co-registration for automated segmental and total liver T1/T2 quantification at 1.5 and 3 T [37]. Such approaches may inform future low-field analysis workflows, but do not validate reconstruction-related quantitative accuracy at 0.4 T.

This study has limitations. First, the design was cross-sectional, with different animals at each timepoint, so disease-stage effects and pathology-specific effects cannot be fully separated. Second, exact voxel-level co-registration between the three MRI regions of interest and whole-section histology was not possible, although tissue sampling was targeted to the central slice. Third, high inflammation and fibrosis thresholds were cohort-specific operating points and should not be interpreted as clinical cutoffs. Fourth, the model did not reproduce the full heterogeneity of human MASLD or iron-overloaded phenotypes. Fifth, the FcT1 model assumed a fixed fat T1 of 200 ms. Sixth, the magnitude-based fat-fraction model did not include voxel-wise off-resonance correction, so PDFF is an apparent estimate and residual bias could not be quantified retrospectively from the retained magnitude data; this uncertainty may propagate into FcT1. Seventh, AIRS was applied only to T1, and paired AIRS ON/OFF data were unavailable in diseased animals. Finally, accessibility, cost-effectiveness, and serial patient monitoring were not tested directly and should be considered translational motivations rather than findings of this animal study.

## Conclusion

In conclusion, multiparametric 0.4-T MRI with deep-learning-reconstructed T1 mapping showed complementary, partly stage-dependent associations with MASLD pathology in rats. T2 and PDFF primarily tracked steatosis, T2^*^ retained an inflammation-related association after stage adjustment, and T1 elevation characterized active high-burden progression rather than isolated residual fibrosis. T1 values were reconstruction-dependent, and PDFF should be interpreted as an apparent estimate. These findings support further platform-calibrated human validation of low-field multiparametric MRI for MASLD phenotyping.

## Supplementary information

**Additional file 1: Supplementary Figure S1.** Incremental contribution of hepatic iron (Fe) to quantitative MRI metrics. For each MRI metric, bars show the change in model R² (coefficient of determination) when adding Fe to a base linear model that includes timepoint/group (ΔR² = R²_{group+Fe} − R²_{group}). Thus, ΔR² quantifies the unique variance explained by Fe beyond timepoint/group. Multiple testing was controlled using the Benjamini–Hochberg FDR procedure; significance was defined by FDR-adjusted q values (qBH < 0.05). No Fe effects reached significance in this dataset (all bars “Not Sig”). **Supplementary Figure S2.** Representative histology images across experimental groups. Representative histologic sections are shown for selected experimental timepoint/groups (0d, 2d, 4d, 7d, 14d, 28d, 63d, F4N20, and F9N15) to illustrate stage-dependent changes during MASLD induction and recovery. Scale bars indicate 50 μm for H & E, Fe, and CD68 immunostaining, and 200 μm for Sirius Red staining. **Supplementary Figure S3.** Representative MRI images across experimental groups. Representative axial quantitative MRI parametric maps of the liver are shown across experimental timepoint/groups (0d, 2d, 4d, 7d, 14d, 28d, 63d, F4N20, and F9N15). Rows display native T1, T2, PDFF, and T2* maps, respectively. These representative images illustrate stage-dependent changes during MASLD induction and recovery. **Supplementary Table S1.** Screening Analysis of Pathological Determinants: Incremental Explained Variance (ΔR²) and Change in Cross-Validated Error (ΔCV_NRMSE). **Supplementary Table S2.** Standardized Multivariable Regression Identifying Pathological Determinants of Quantitative MRI Metrics. **Supplementary Table S3.** Spearman Partial Correlations Between Quantitative MRI Metrics and Histopathological Metrics Adjusted for Hepatic Iron Deposition (Fe). **Supplementary Table S4.** Internal Validation of Diagnostic Performance Using Bootstrap Resampling with Out-of-Bag Evaluation. **Supplementary Table S5.** Internal Validation of Diagnostic Performance Using Repeated Stratified 10-Fold Cross-Validation. **Supplementary Table S6.** Leave-One-Timepoint-Out Cross-Validation for Discriminating High Inflammation (CD68 > 2.33%): Fold-Level Results for T2 and T2*. **Supplementary Table S7.** Leave-One-Timepoint-Out Cross-Validation for Discriminating High Inflammation (CD68 > 2.33%): Summary Across Splits. **Supplementary Table S8.** Leave-One-Timepoint-Out Cross-Validation for Discriminating High Fibrosis (CPA > 1.95%, > 2.42%, and > 2.90%): Fold-Level Results. **Supplementary Table S9.** Leave-One-Timepoint-Out Cross-Validation for Discriminating High Fibrosis (CPA > 1.95%, > 2.42%, and > 2.90%): Summary Across Splits. **Supplementary Table S10.** GroupKFold Validation for Discriminating High Inflammation (CD68 > 2.33%): Fold-Level Results. **Supplementary Table S11.** GroupKFold Validation for Discriminating High Inflammation (CD68 > 2.33%): Summary Across Folds. **Supplementary Table S12.** GroupKFold Validation for Discriminating High Fibrosis (CPA > 1.95%, > 2.42%, and > 2.90%): Fold-Level Results. **Supplementary Table S13.** GroupKFold Validation for Discriminating High Fibrosis (CPA > 1.95%, > 2.42%, and > 2.90%): Summary Across Folds. **Supplementary Table S14.** Spearman Partial Correlations Between MRI Metrics and Pathology Metrics Adjusted for Hepatic Iron and Timepoint. **Supplementary Table S15.** Standardized Multivariable Regression Coefficients for Pathology Determinants of MRI Metrics Adjusted for Timepoint. **Supplementary Table S16.** Timepoint (Group) Terms From Standardized Multivariable Regression Models. **Supplementary Table S17.** Variance inflation factors for pathology predictors.

## Data Availability

The de-identified datasets and analytic code supporting the conclusions of this article are available from the corresponding author upon reasonable request.

## Abbreviations

AUROC: Area under the receiver operating characteristic curve
CD68: CD68-positive area fraction
CPA: Collagen proportionate area
FcT1: Fat-corrected T1
Fe: Perls’ Prussian blue-positive area fraction
LV: Lipid vacuole area fraction
MASLD: Metabolic dysfunction-associated steatotic liver disease
MRI: Magnetic resonance imaging
PDFF: Proton density fat fraction (reported as an apparent estimate in this study)
VIF: Variance inflation factor
