# Supplementary material for: Multiparametric 0.4-T low-field MRI mapping for metabolic dysfunction-associated steatotic liver disease in rats
Source: Eur Radiol Exp. 2026 Nov 14;10:185. doi: 10.1186/s41747-026-00852-6 (PMC13769794; doi:10.1186/s41747-026-00852-6)
Supplement: Supplementary file 1 [file 41747_2026_852_MOESM1_ESM.pdf]

Multiparametric 0.4-T low-field MRI mapping for metabolic dysfunction-associated steatotic liver disease in rats

ELECTRONIC SUPPLEMENTARY MATERIAL

Animals and husbandry

Male Wistar rats were purchased at 4 weeks of age (Japan SLC, Shizuoka, Japan) and acclimatized for 1 week before study initiation. The choline-deficient, L-amino acid–defined, high-fat diet (CDAHFD) was obtained from Research Diets, Inc. (New Brunswick, NJ, USA; product no. A16092003). Animals were acclimatized and housed in the animal facility, and MRI acquisitions were performed in the MRI suite. Animals were housed 2-3 per cage under standard laboratory conditions (temperature 22 ± 2°C, 12-h light/dark cycle) with ad libitum access to water and the assigned diet (CDAHFD or normal chow). Animals were allocated to prespecified endpoint (cross-sectional; no repeated measures) without randomization and monitored at least daily for general health. Two rats allocated to the Day 63 group died spontaneously (suspected liver failure).

Blinding

Blinding was not feasible during group allocation and experiment conduct: the investigator responsible for allocation, personnel preparing the diets (CDAHFD vs chow), and MRI operators were aware of group assignment. During data entry and final statistical analysis, animals were identified by coded IDs and group labels were masked until the primary analyses were completed. Outcome assessment (ROI-based MRI measurements and digital pathology quantification) was performed using coded IDs with group allocation concealed.

Anesthesia and euthanasia

For MRI, anesthesia was induced by intraperitoneal three-drug mixed anesthesia (medetomidine/midazolam/butorphanol; 0.15/2.0/2.5 mg/kg). Spontaneous breathing was maintained during scanning, and body temperature was supported with external warming. At the endpoint, rats were euthanized by CO2 inhalation; death was confirmed prior to tissue collection.

Inclusion/Exclusion criteria

Inclusion and exclusion criteria were established a priori. Animals were included if they completed the assigned protocol and scheduled MRI and tissue collection. Animals were excluded only if they died or met humane endpoints before completion; preplanned reserve animals (defined at enrollment) were not included once the prespecified group sizes were met. No other animals or data points were excluded.

Humane endpoints / welfare

Animals were monitored at least daily for general health. Steps to reduce pain and distress included general anesthesia during MRI with medetomidine/midazolam/butorphanol, with butorphanol included for analgesia, physiologic monitoring during MRI, and maintenance of body temperature. Humane endpoints were predefined for growing animals: (i) growth retardation (body weight <80% of the mean weight of the age-matched experimental group) and (ii) failure to thrive (absence of weight gain or weight loss for 3 consecutive days during the active growth phase); animals meeting humane endpoint criteria would be euthanized.

Housing/Enrichment

No environmental enrichment was provided.

MRI acquisition and quantitative mapping

All MRI examinations were performed on a 0.4-T open MRI system (Gravity MRI, FUJIFILM Healthcare). To minimize potential confounders, MRI acquisitions were performed under a standardized protocol and, when feasible, the acquisition order was interleaved across experimental groups/timepoints. Quantitative MRI metrics included native T1, fat-corrected T1 (FcT1), T2, T2*, and an apparent proton density fat fraction (PDFF) estimate. For T1 mapping, SwiftMR (version [3.0.13.1]; AIRS Medical Inc., Seoul, South Korea) was applied to standard-reconstructed DICOM images (image-domain deep-learning reconstruction) (1,2). Other sequences (T2, T2*, and apparent PDFF) used the system's standard reconstruction.

Sequence parameters

Key acquisition parameters are summarized in Table.

**Parameter**	**T1/FcT1**	**T2**	**T2*/apparent PDFF**
Sequence	2D IR-FSE (FIR)	Two-echo FSE	Four-echo mGRE
Imaging plane	Axial	Axial	Axial
TR (ms)	2000	2000	250
TI (ms)	600	N/A	N/A

**Parameter**	**T1/FcT1**	**T2**	**T2*/apparent PDFF**
TE (ms)	15.0	15.0/60.0	8.9/17.8/26.7/35.6
Flip angle (°)	90	90	12
FOV (mm)	100	100	100
Acquisition matrix	256 × 128	256 × 128	240 × 128
Reconstruction matrix	256 × 256	256 × 256	256 × 256
Number of acquired slices	3	3	3
Slice thickness (mm)	8.0	8.0	8.0
Slice interval (mm)	9.0	9.0	9.0
Inter-slice gap (mm)	1.0	1.0	1.0
Receiver bandwidth (kHz)	34.8	34.8	30.0
NEX	2	2	4

**Parameter**	**T1/FcT1**	**T2**	**T2*/apparent PDFF**
Acquisition time per sequence (min:s)	2:08	2:50	2:09
Echo-train length	4	3	N/A

Note.—For T1/FcT1 calculation, the first-echo image of the two-echo FSE sequence (TE = 15.0 ms) served as the TI = 0-ms reference, and the 2D IR-FSE sequence provided the TI = 600-ms image; no separate TI = 0-ms IR-FSE acquisition was performed. **Abbreviations**: FSE = fast spin-echo; IR = inversion recovery; mGRE = multi-echo gradient-echo; PDFF = proton density fat fraction (reported as an apparent estimate in this study); FcT1 = fat-corrected T1; T1 = longitudinal relaxation time; T2 = transverse relaxation time; T2* = apparent transverse relaxation time.

Native T1 mapping (two-point IR) (3)

Acquisition details. T1/FcT1 mapping combined a 2D IR-FSE acquisition (FIR; TR = 2000 ms, TI = 600 ms, TE = 15.0 ms, echo-train length = 4) with the first-echo image of the two-echo FSE acquisition (TE = 15.0 ms), which served as the TI = 0-ms reference. Both sequences were acquired axially with an FOV of 100 mm, acquisition matrix 256 × 128, reconstruction matrix 256 × 256, three 8.0-mm slices, a 9.0-mm slice interval (1.0-mm inter-slice gap), and NEX = 2. Receiver bandwidth was 34.8 kHz. Acquisition times were 2:08 for the 2D IR-FSE

sequence and 2:50 for the two-echo FSE sequence. No separate TI = 0-ms IR-FSE acquisition was performed.

Under an ideal inversion-recovery model, the longitudinal magnetization at inversion time TI is:

Equation (S1) $$M(TI) = M0 \times [1 - 2 \times \exp(-TI / T1)]$$

Assuming M0 is given by the TI = 0 magnitude image, T1 can be computed from the TI = 600 ms measurement as:

Equation (S2) $$T1 = -TI / \ln\{(1 - M(TI)/M0) / 2\}$$

In this implementation, the first-echo image of the two-echo FSE acquisition (TE = 15.0 ms) served as the positive TI = 0-ms (M0) reference, and the 2D IR-FSE image at TI = 600 ms provided the inversion-recovery measurement. The TI = 600-ms image was selected to ensure a positive signal, thereby avoiding sign ambiguity and instability around the null point when using magnitude-only inversion-recovery data.

Fat-corrected T1 (FcT1) using an IR-consistent exponential mixing model

FcT1 was computed using the apparent PDFF estimate in a two-component inversion-recovery model. Because the inversion-recovery signal from a voxel containing both water and fat is a weighted sum of exponentials, FcT1 was derived using an IR-consistent exponential mixing model rather than a linear relaxation-rate (R1) mixing approximation.

Let f denote voxel-wise apparent fat fraction (f = PDFF/100) and (1 − f) the water fraction. Let T1w be the water-specific T1 (FcT1) and T1f the T1 of the fat component (fixed to 200 ms). The normalized inversion-recovery signal can be written as:

Equation (S3) $m(TI) = 1 - 2 \times [(1 - f) \times \exp(-TI / T1w) + f \times \exp(-TI / T1f)]$

Solving Equation (S3) for T1w yields:

Equation (S4) $\exp(-TI / T1w) = \{[(1 - m(TI)) / 2] - f \times \exp(-TI / T1f)\} / (1 - f)$

Taking the natural logarithm of Equation (S4) and solving for T1w gives:

Equation (S5) $FcT1\ (= T1w) = -TI / \ln\{ \{[(1 - m(TI)) / 2] - f \times \exp(-TI / T1f)\} / (1 - f) \}$

This exponential mixing formulation is consistent with inversion-recovery physics and is particularly important at low field, where liver T1 is shorter and TI/T1 can be large (e.g., TI = 600 ms with liver T1 on the order of a few hundred milliseconds). To combine the apparent PDFF estimate with the two-point IR data for FcT1 estimation, inter-sequence motion was assumed to be minimal under three-drug anesthesia (see above). Scans were performed without respiratory gating while maintaining spontaneous breathing. To mitigate partial-volume effects and potential misregistration, ROI-based analyses were restricted to the central liver slice.

Fixing T1f = 200 ms is consistent with prior in vivo measurements reporting subcutaneous fat T1 on the order of ~0.2 s and minimal field-strength

dependence across 1.4–4.7 kgauss (0.14–0.47 T), which brackets 0.4 T (3); however, because hepatic fat T1 in this specific animal model at 0.4 T was not directly measured, this choice should be interpreted as a simplifying assumption and the impact of alternative T1f values can be evaluated in a sensitivity analysis (e.g., 180/200/220 ms).

T2 mapping

T2 maps were generated from a multi-echo fast spin-echo (FSE) acquisition. For each voxel, magnitude signal intensity as a function of echo time (TE) was modeled using a mono-exponential decay:

Equation (S6) $I(TE) = I0 \times \exp(-TE / T2)$

A voxel-wise mono-exponential model yielded the T2 map (T2map). In this study, two echoes were acquired (TE = 15 and 60 ms; echo spacing = 45 ms) to reduce acquisition time. At 0.4 T, reduced susceptibility effects and improved RF homogeneity may mitigate nonideal contributions to decay(3). T2 was estimated from the two-echo signal ratio under the mono-exponential model (Eq. S6) and reported as an apparent T2.

T2 mapping and apparent PDFF estimation*

T2* and apparent PDFF were estimated from a multi-echo gradient-echo (mGRE) acquisition with a low flip angle to minimize T1 weighting. The first two

echoes (TE = 8.9 and 17.8 ms) corresponded approximately to out-of-phase and in-phase conditions at 0.4 T. Parametric maps for T2* and apparent PDFF were generated using an in-house program (MRMGRE_map; implemented with MATLAB Runtime [R2020b]; The MathWorks, Natick, MA, USA), based on a previously reported MRM-GRE method.

Water and fat signals were modeled as decaying exponentials:

Equation (S7a) $I_w(TE) = I_{w0} \times \exp(-TE / T2_w^*)$

Equation (S7b) $I_f(TE) = I_{f0} \times \exp(-TE / T2_f^*)$

Using a simplified two-component magnitude model, the combined signal can be approximated as:

Equation (S7c) $I(TE) \approx \mathrm{sqrt}(I_{w0}^2 + I_{f0}^2 + 2 \times I_{w0} \times I_{f0} \times \cos(\Delta\omega \times TE)) \times \exp(-TE / T2^*)$

where $\Delta\omega$ is the fixed nominal chemical-shift angular frequency difference between water and fat at 0.4 T, and T2* is the apparent transverse relaxation time. Voxel-wise fitting of multi-echo magnitude data provided estimates of Iw0, If0, and T2*.

Apparent PDFF was then calculated as:

Equation (S8) $\mathrm{PDFF}(\%) = 100 \times [I_{f0} / (I_{w0} + I_{f0})]$

The fitting used magnitude data and did not include a voxel-wise B0 field map, phase-based water–fat separation, or off-resonance correction. Consequently, the reported PDFF values are apparent estimates rather than fully confounder-corrected PDFF. The direction and magnitude of

residual off-resonance bias cannot be quantified retrospectively from the retained magnitude images alone.

Image analysis and region-of-interest measurements

Quantitative maps (T1, FcT1, T2, T2*, and PDFF) were generated on a voxel-wise basis. For each animal, liver measurements were extracted from regions of interest (ROIs) drawn on parametric maps. ROIs were placed to avoid large vessels and visible artifacts; the mean value across ROIs was used for analysis. ROI placement was performed by a radiologic technologist with 7 years of experience. ROI placement strategy and software. For each animal, measurements were performed on a single central liver slice. Three circular ROIs (10 mm^2 each) were placed within liver parenchyma while avoiding large vessels, the gallbladder, and visible artifacts; the mean across the three ROIs was used for analysis. ROIs were propagated across all parametric maps. When SwiftMR increased the reconstructed matrix size (e.g., to 1024 × 1024), ROIs were redrawn at the corresponding anatomical locations to match the other sequences as closely as possible. ROI analysis was performed using EV Insite R (version [3.10.3.3]; PSP Corporation, Tokyo, Japan). ROI reproducibility (precision of ROI-based measurements).

ROI reproducibility / reliability

To assess the precision of ROI-based quantitative measurements, ROI placement and extraction were repeated in 43 animals selected from all 9

experimental groups to cover the full dynamic range of signal intensities. For intraobserver reliability, the primary observer repeated ROI placement and measurement after a 4-week washout interval. For interobserver reliability, a second reader independently performed ROI placement and measurement using the same protocol. Both observers were blinded to group allocation and to prior measurements. Reproducibility was assessed for all ROI-derived parameters (T1, fat-corrected T1 [FcT1], T2, T2*, and PDFF). Agreement was quantified using the intraclass correlation coefficient (ICC) with 95% confidence intervals, using a two-way mixed-effects model for absolute agreement and single measurements (ICC[A,1]). Measurement error was further characterized using Bland–Altman analysis (bias and 95% limits of agreement). Differences were defined as repeat − initial (intraobserver) and reader 2 − reader 1 (interobserver). The within-subject standard deviation (wSD), within-subject coefficient of variation (wCV), and 95% repeatability coefficient (RC95) were calculated as:

wSD = SD(diff)/√2; wCV = 100 × wSD / mean(paired means); and RC95 = 1.96 × SD(diff) (equivalently, RC95 = 2.77 × wSD).

Histologic section preparation and staining

After CO_2 euthanasia, to minimize spatial mismatch with MRI, tissue blocks for whole-slide analysis were sampled from liver regions corresponding as closely as feasible to the central slice used for MRI ROI measurements. The entire liver was removed and rinsed in phosphate-buffered saline (PBS; Gibco, Thermo Fisher

Scientific, Waltham, MA) to clear excess blood. Each lobe was weighed, examined for gross lesions, and fixed in 10% neutral buffered formalin (FUJIFILM Wako Pure Chemical Corp., Osaka, Japan) for 48 hours. Once fixed, tissues were dehydrated through ascending ethanol concentrations (70%-100%), cleared in xylene (FUJIFILM Wako), and embedded in Histosec (Sigma-Aldrich, Germany). Sections (4-5 μm) were cut using a Leica RM2235 microtome (Leica Biosystems) and mounted on glass slides (CRE-01; Matsunami Glass, Osaka, Japan).

For hematoxylin and eosin (H&E) staining, sections were deparaffinized in xylene, rehydrated in descending ethanols, and stained with Mayer's hematoxylin for 5 minutes, according to a previously reported protocol. Sections were rinsed in tap water, briefly dipped in 1% acid alcohol (1% HCl in 70% ethanol), rinsed again, and counterstained with eosin (Muto Pure Chemicals, Tokyo, Japan) for 10 minutes.

Sirius Red staining was carried out on adjacent sections using a 0.1% Picro Sirius Red F3BA solution (Waldeck GmbH & Co. KG, Munster, Germany) in saturated picric acid (FUJIFILM Wako) for 60 minutes. Slides were rinsed in acidified water (0.5% glacial acetic acid), dehydrated in ethanol, cleared in xylene, and mounted with Entellan New (Sigma-Aldrich).

Iron content was assessed by Perls' Prussian blue (Berlin blue) staining. Sections were immersed in a 1:1 mixture of 2% potassium ferrocyanide and 1% hydrochloric acid for 30 minutes, rinsed thoroughly in distilled water, and counterstained with nuclear fast red (Abcam) for 5 minutes.

For immunohistochemistry, slides were deparaffinized, rehydrated, and subjected to antigen retrieval in citrate buffer (pH 6.0) at 95 C for 20 minutes. Endogenous peroxidase activity was blocked in methanol containing 0.3% H_2O_2 for 15 minutes. Primary antibodies were applied overnight at 4 C: anti-CD68 (clone ED1, Cat. #MCA341, Bio-Rad, Hercules, CA) at 1:200. After washing in PBS, slides were incubated for 30 minutes with a polymer-based secondary antibody system (Histofine Simple Stain Rat MAX PO, Nichirei Biosciences, Tokyo, Japan), visualized using 3,3′-diaminobenzidine (DAB; Dako Agilent, Santa Clara, CA), and counterstained with hematoxylin.

Whole-slide imaging and digital pathology quantification

Whole-slide images were digitized and analyzed in QuPath (version 0.5.1). Steatosis was quantified as lipid vacuole area fraction (LV%), fibrosis as collagen proportionate area (CPA%), inflammation as CD68-positive area fraction, and iron deposition as a Perls stain-based hepatic iron area proportion (Fe%). For each stain, tissue was detected automatically and the analysis was restricted to liver parenchyma. Slides were digitized using a NanoZoomer-XR Digital slide scanner C12000 (Hamamatsu Photonics, Japan) with a 20× objective (pixel size, 0.44 μm/px). In QuPath, stain-specific parameter settings were defined by the detection threshold (threshold), stain vectors (r, g, b) used for color deconvolution, and analysis resolution (pixel size used for detection). Unless

otherwise noted, analyses were performed at full resolution (0.44 μm/px); for lipid vacuole (fat) quantification on H&E, the analysis resolution was intentionally downsampled to 0.88 μm/px to reduce false detection of small optically clear structures (e.g., sinusoids). Parameter settings were as follows: fat: threshold 0.10; stain vector r = 0.3155105736760095, g = -0.5981592040221263, b = 0.736653680193245; analysis resolution 0.88 μm/px. Sirius Red: threshold 0.07; stain vector r = 0.8048893353240665, g = 0.514929202101732, b = 0.29495944586409883; analysis resolution full resolution (0.44 μm/px). Perls (Fe): threshold 0.09; stain vector r = 0.8650665627080952, g = 0.456038187958662, b = 0.20901916947443105; analysis resolution full resolution (0.44 μm/px). CD68: threshold 0.28; stain vector r = 0.26916687204956063, g = 0.5682411743268502, b = 0.777593185920953; analysis resolution full resolution (0.44 μm/px). LV% (steatosis): On H&E slides, lipid vacuoles were segmented as optically clear intraparenchymal spaces using an automated approach (e.g., thresholding or a trained pixel classifier). LV% was calculated as 100 × (lipid vacuole area / total tissue area). CPA% (fibrosis): On Sirius Red slides, collagen-positive pixels were identified by color deconvolution and thresholding. CPA% was calculated as 100 × (collagen-positive area / total tissue area). CD68-positive area fraction (inflammation): On CD68 immunostained slides, DAB-positive pixels were detected using optical density thresholding. The CD68-positive area fraction was calculated as 100 × (CD68-positive area / total tissue area). Fe (hepatic iron area fraction): On Perls-stained slides, iron-positive (blue) pixels were identified by color thresholding. A

quantitative iron area fraction was computed from the proportion of iron-positive pixels within tissue.

Sample size considerations

No formal a priori power calculation was performed because effect sizes for low-field FcT1 in this animal model were not available at study design. The prespecified analysis set (N = 90) was targeted to support stable multivariable regression across coexisting histopathological components (Fe%, LV%, CPA%, and CD68%), providing 22.5 observations per predictor and exceeding commonly used rules of thumb for model stability, while spanning acute, progression, and recovery phases. Baseline and acute groups (Day 0/2/4) were set at n = 6 because these cohorts were expected to be relatively homogeneous and intended to establish reference values, whereas progression and recovery groups were set at n = 12 to accommodate increasing biological variability and to support multivariable and ROC analyses with resampling-based internal validation while minimizing animal use under the 3R principles (Reduction). Uncertainty was addressed by reporting effect sizes with 95% confidence intervals and by performing internal validation (bootstrap and cross-validation), including group-aware validation by experimental timepoint/group as described below.

Internal validation of ROC analyses

Internal validation (general procedure)

Internal validation was performed to assess the robustness of ROC-based discrimination within this cohort. Across all validation procedures, the ROC direction ("positive rule"; ≥ cutoff vs ≤ cutoff) and the Youden-optimal cutoff were determined only in the training set and then applied unchanged to the held-out test set to avoid optimistic bias. Test-set AUC was computed only when the held-out test set contained both outcome classes; the number of valid resamples/folds is reported as *n_valid_auc*.

Bootstrap resampling with out-of-bag evaluation

Bootstrap resampling was performed at the animal level (B = 2000). For each bootstrap replicate, a bootstrap sample (training set) was drawn with replacement from the full dataset (N = 90), and animals not sampled constituted the out-of-bag (OOB) test set. The ROC curve was fitted in the training set, the optimal cutoff was selected using the Youden index, and the cutoff and ROC direction were then applied to the OOB set to compute sensitivity, specificity, accuracy, and AUC (when both classes were present in the OOB set). Performance metrics are summarized as median [2.5th–97.5th percentiles] across replicates.

Repeated stratified 10-fold cross-validation

Repeated stratified 10-fold cross-validation (10 folds × 50 repeats; total 500 test folds) was performed at the animal level, with stratification by outcome class (high vs low). In each split, the ROC curve and Youden-optimal cutoff were determined in the training folds and evaluated in the held-out fold using the same procedure as above. AUC was computed only when the held-out fold contained both outcome classes. Performance metrics are summarized as median [2.5th–97.5th percentiles] across folds.

Group-aware validation by experimental timepoint/group.

To account for clustering by experimental timepoint/group, group-aware validation was performed using:

(1) leave-one-timepoint-out (LOGO) validation, treating each experimental timepoint/group (Day0, Day2, Day4, Day7, Day14, Day28, Day63, F4N20, F9N15) as one held-out test group; and

(2) timepoint-grouped k-fold cross-validation (GroupKFold; k = 3), assigning whole timepoint/groups to folds so that no group contributed animals to both training and test sets within a split. For each split, the Youden-optimal cutoff and ROC direction were determined in the training set and then evaluated on the held-out group fold. Test-set AUC was computed only when the held-out set contained both outcome classes.

Software implementation and reproducibility

All analyses were performed in R (version 4.4.2). ROC analyses and resampling-based internal validation were implemented using pROC and rsample, with supporting packages readxl, dplyr, tibble, purrr, and writexl (package versions are provided in sessionInfo()). Bootstrap internal validation used B = 2000 resamples with out-of-bag evaluation (minimum out-of-bag size = 5), and repeated stratified cross-validation used 10 folds × 50 repeats. Random seeds were fixed for reproducibility (bootstrap: SEED_BOOT_BASE = 1000; cross-validation: SEED_CV_BASE = 2000; incremented by analysis configuration).

Supplementary Results

S1. Incremental contribution of hepatic iron (Fe) to MRI signal variance

When hepatic iron (Fe) was added to models already adjusted for experimental timepoint/group, the incremental explained variance (ΔR^2) was small across all MRI metrics. The largest ΔR^2 was observed for T2* (1.07×10^{-3}) and T2 (4.98×10^{-4}), whereas ΔR^2 was negligible for T1 (4.20×10^{-7}), FcT1 (3.22×10^{-5}), and PDFF (1.70×10^{-5}). None of these incremental contributions remained significant after false discovery rate correction (all $q_{BH} > .05$), indicating that Fe explained little additional variance beyond stage/timepoint effects in this dataset (Supplementary Figure S1).

S2. Detailed Fe-adjusted partial correlations (Figure 3, Table 3)

In Spearman partial correlation analyses adjusting for Fe, steatosis (LV) showed very strong positive correlations with T2 (ρ = 0.90, P < .001) and PDFF (ρ = 0.88, P < .001), and a moderate positive correlation with T2* (ρ = 0.36, P < .001). Native T1 was inversely correlated with LV (ρ = −0.48, P < .001), whereas FcT1 showed a modest positive correlation with LV (ρ = 0.34, P = .001). Inflammation (CD68) correlated strongly with T2 (ρ = 0.68, P < .001) and T2* (ρ = 0.64, P < .001), and moderately with FcT1 (ρ = 0.37, P < .001) and PDFF (ρ = 0.48, P < .001). Fibrosis (CPA) showed positive correlations with native T1 (ρ = 0.32, P = .002) and FcT1 (ρ = 0.24, P = .02), but was not significantly associated with T2 (ρ = 0.12, P = .27) or T2* (ρ = 0.09, P = .40). The correlation between PDFF and CPA was weakly negative and did not reach significance (ρ = −0.17, P = .11) (Figure 3, Table 3).

S3. Full standardized multivariable regression results (Supplementary Table S2)

In standardized multivariable models simultaneously including Fe, CPA, LV, and CD68, the pathological determinants of MRI signals differed across metrics (Supplementary Table S2). For FcT1, CPA was the largest positive determinant (β = 0.833, P < .001), whereas Fe contributed negatively (β = −0.377, P < .001). Similarly, for native T1, CPA had the largest positive contribution (β = 0.934, P <

.001), while LV (β = −0.416, P < .001) and Fe (β = −0.390, P < .001) were negative determinants. For T2*, inflammation (CD68) was the strongest positive determinant (β = 0.541, P < .001), with an additional independent contribution from CPA (β = 0.313, P = .005) and a negative contribution from Fe (β = −0.340, P < .001). For T2, LV was the dominant determinant (β = 0.910, P < .001), with a smaller positive contribution from CD68 (β = 0.094, P = .047). PDFF was explained almost entirely by LV (β = 0.998, P < .001); CPA contributed independently in the negative direction (β = −0.157, P < .001), while Fe and CD68 were not significant.

S4. ROI measurements demonstrated excellent reproducibility.

Intraobserver ICCs were 0.99–1.00 across parameters, and interobserver ICCs were 0.95–1.00, indicating excellent agreement. Bland–Altman analysis showed minimal systematic bias (range, −0.70 to 0.19), with narrow 95% limits of agreement. Precision metrics were similarly favorable, with wCV values of 1.0%–2.4% across parameters.

Supplementary Figures

Supplementary Figure S1. Incremental contribution of hepatic iron (Fe) to quantitative MRI metrics.

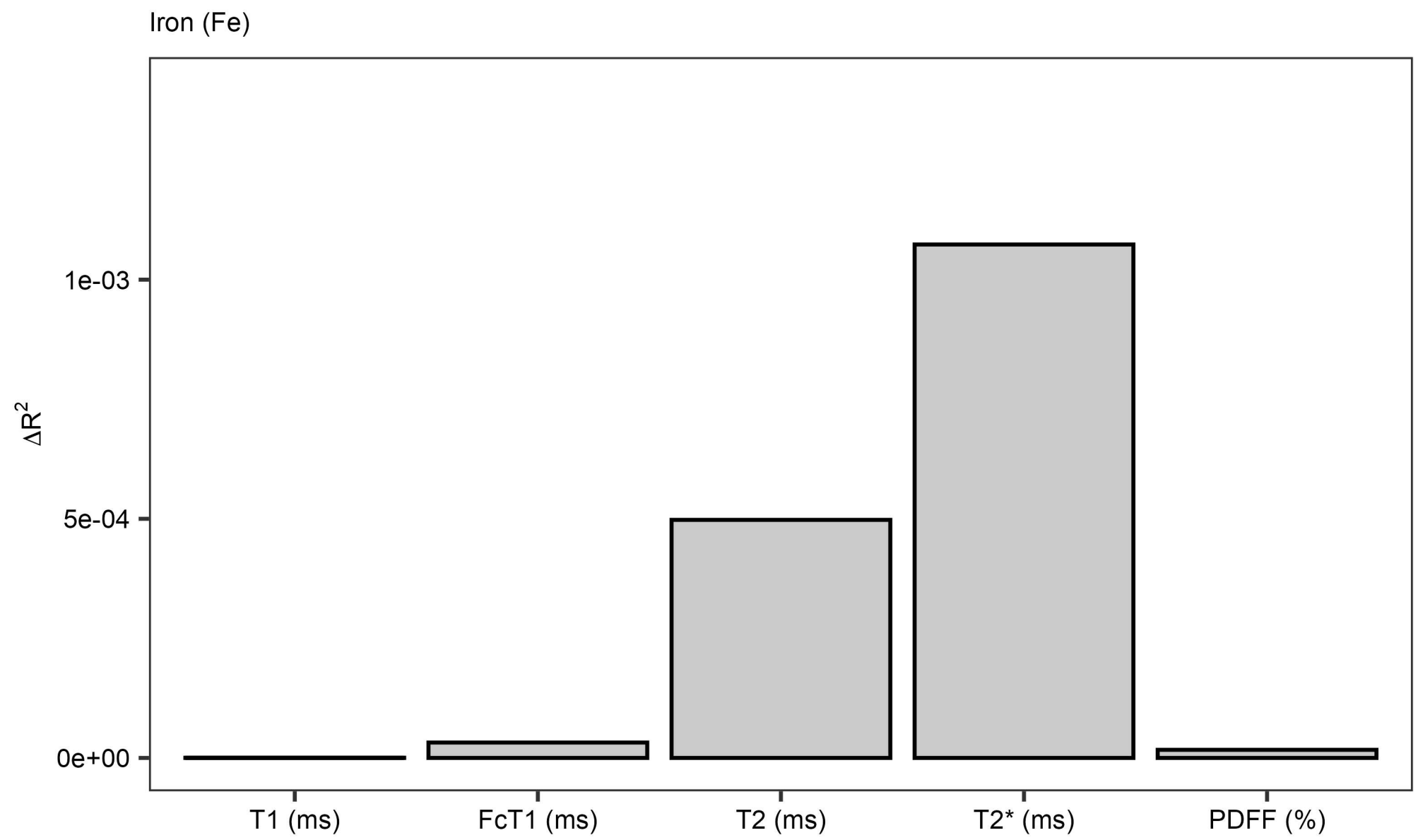

For each MRI metric, bars show the change in model R^2 (coefficient of determination) when adding Fe to a base linear model that includes timepoint/group ($\Delta R^2 = R^2_{group+Fe} - R^2_{group}$). Thus, ΔR^2 quantifies the unique variance explained by Fe beyond timepoint/group. Multiple testing was controlled using the Benjamini–Hochberg FDR procedure; significance was defined by FDR-adjusted q values (qBH < 0.05). No Fe effects reached significance in this dataset (all bars “Not Sig”).

Supplementary Figure S2. Representative histology images across experimental groups.

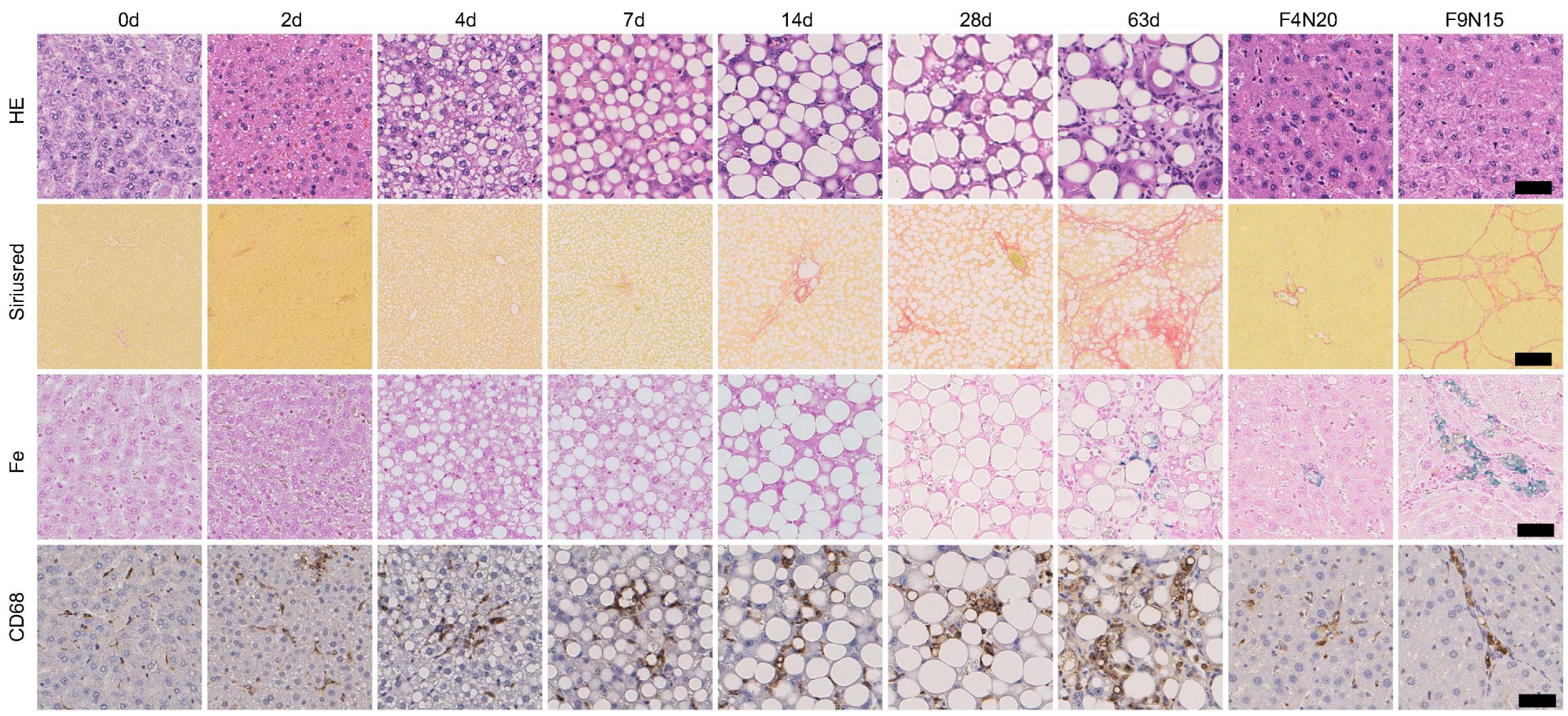

Representative histologic sections are shown for selected experimental timepoint/groups (0d, 2d, 4d, 7d, 14d, 28d, 63d, F4N20, and F9N15) to illustrate stage-dependent changes during MASLD induction and recovery. Scale bars indicate 50 µm for H&E, Fe, and CD68 immunostaining, and 200 µm for Sirius Red staining. **Abbreviations:** d = days; HE (H&E) = hematoxylin and eosin; Sirius Red = Picro Sirius Red staining; Fe = iron deposition (Perls' Prussian blue staining); CD68 = CD68 immunostaining (macrophage marker); MASLD = metabolic dysfunction–associated steatotic liver disease; F4N20 and F9N15 = recovery groups (see study design for definitions).

Supplementary Figure S3. Representative MRI images across experimental groups.

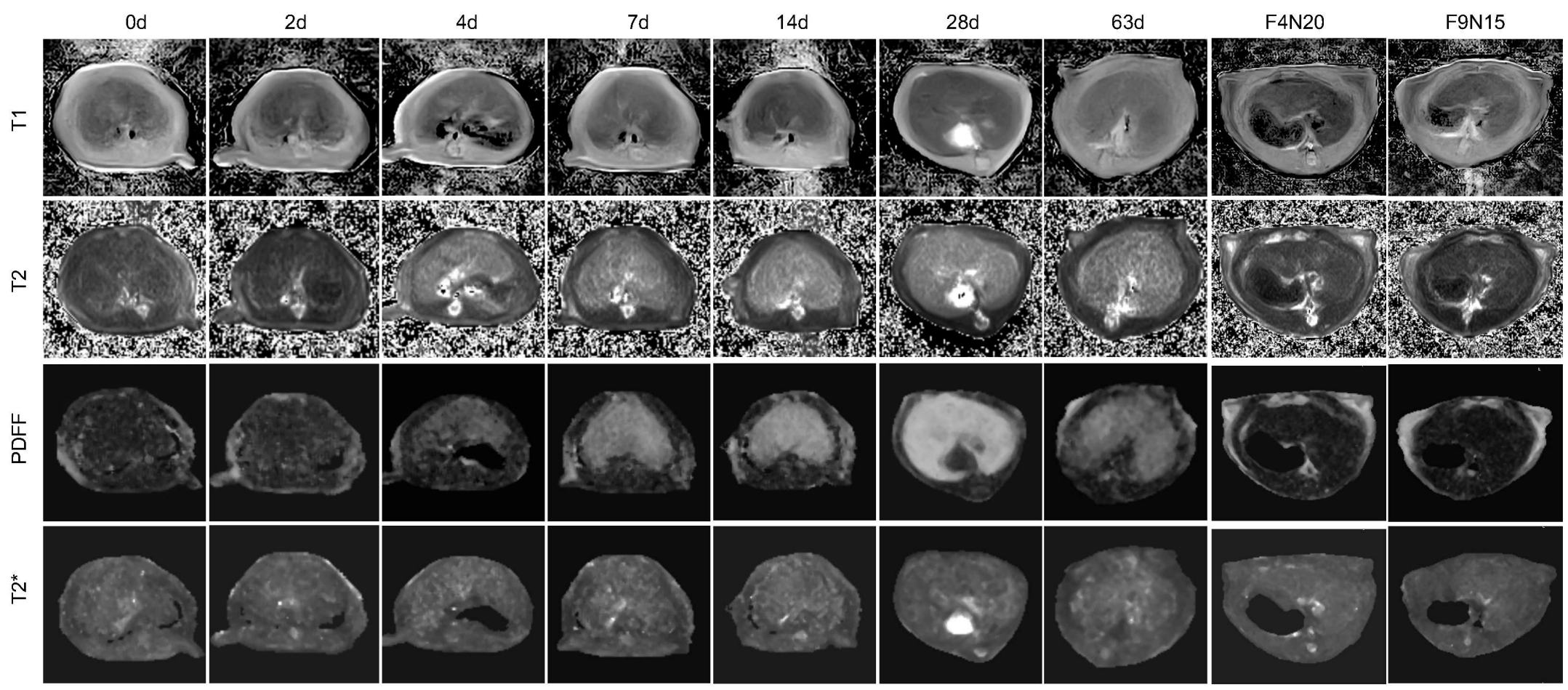

Representative axial quantitative MRI parametric maps of the liver are shown across experimental timepoint/groups (0d, 2d, 4d, 7d, 14d, 28d, 63d, F4N20, and F9N15). Rows display native T1, T2, PDFF, and T2* maps, respectively. These representative images illustrate stage-dependent changes during MASLD induction and recovery. **Abbreviations:** d = days; MASLD = metabolic dysfunction–associated steatotic liver disease; MRI = magnetic resonance imaging; PDFF = proton density fat fraction; T1 = longitudinal relaxation time; T2 = transverse relaxation time; T2* = apparent transverse relaxation time; F4N20 and F9N15 = recovery timepoint/groups (see study design for definitions).

Supplementary Tables

Supplementary Table S1. Screening Analysis of Pathological Determinants: Incremental Explained Variance (ΔR²) and Change in Cross-Validated Error (ΔCV_NRMSE)

MRI Metric	Added Pathology	ΔR^2	ΔCV NRMSE	P Value	q Value (FDR)
T1	Iron (Fe)	4.2×10^{-7}	0.004	.98	.98
	Steatosis (LV)	0.007	0.00033	.01	.06
	Fibrosis (CPA)	0.00035	0.004	.58	.81
	Inflammation (CD68)	0.00061	0.003	.47	.72
FcT1	Iron (Fe)	3.2×10^{-5}	0.003	.86	.91
	Steatosis (LV)	0.002	0.002	.20	.39
	Fibrosis (CPA)	0.003	0.002	.11	.29
	Inflammation (CD68)	0.002	0.004	.21	.39
T2	Iron (Fe)	0.0005	-0.003	.35	.59
	Steatosis (LV)	0.011	-0.01	<.001	<.001
	Fibrosis (CPA)	2.4×10^{-5}	-0.00027	.84	.91
	Inflammation (CD68)	0.00099	-0.002	.19	.39
T2*	Iron (Fe)	0.001	0.002	.61	.81
	Steatosis (LV)	0.011	0.00013	.11	.29
	Fibrosis (CPA)	0.00087	0.00082	.65	.81
	Inflammation (CD68)	0.067	-0.015	<.001	<.001

MRI Metric	Added Pathology	ΔR^2	ΔCV NRMSE	P Value	q Value (FDR)
PDFF	Iron (Fe)	1.7×10^{-5}	0.002	.76	.89
	Steatosis (LV)	0.003	-0.003	<.001	<.001
	Fibrosis (CPA)	0.001	0.002	.005	.03
	Inflammation (CD68)	0.00064	0.002	.05	.18

Note.—For each MRI metric, a base linear model including experimental timepoint/group (categorical) was fit first. Each pathology metric (hepatic iron [Fe%], steatosis [LV%], fibrosis [CPA%], or inflammation [CD68%]) was then added one at a time. ΔR^2 is the incremental variance explained by adding the pathology metric beyond timepoint/group; ΔCV_NRMSE is the corresponding change in 5-fold cross-validated normalized RMSE (negative values indicate lower prediction error). P values are for the added pathology term in the full model; q values are Benjamini–Hochberg false discovery rate–adjusted across all MRI–pathology comparisons in this table. Because pathology metrics evolve with experimental timepoint in this longitudinal model, ΔR^2 in Table S1 reflects incremental information beyond timepoint (i.e., beyond disease stage) and is therefore expected to be attenuated when timepoint captures a large fraction of variance.

Abbreviations: BH = Benjamini–Hochberg; CD68 = CD68-positive area fraction; CPA = collagen proportionate area; CV = cross-validation; FcT1 = fat-corrected T1; FDR = false discovery rate; Fe = hepatic iron area fraction on Perls' Prussian blue stain (Fe%); LV = lipid vacuole area fraction; NRMSE = normalized root mean square error; PDFF = proton density fat fraction; Δ = incremental change versus the base model.

Supplementary Table S2. Standardized Multivariable Regression Identifying Pathological Determinants of Quantitative MRI Metrics

MRI Metric	Predictor	β (Standardized)	Std. Error	P Value	95% CI	Adj. R^2
FcT1	Iron (Fe)	-0.38	0.07	<.001	(-0.51, -0.24)	0.74
	Fibrosis (CPA)	0.83	0.09	<.001	(0.66, 1.01)	
	Steatosis (LV)	0.12	0.08	.12	(-0.03, 0.28)	
	Inflammation (CD68)	0.02	0.10	.82	(-0.18, 0.23)	
T1	Iron (Fe)	-0.39	0.06	<.001	(-0.52, -0.26)	0.76
	Fibrosis (CPA)	0.93	0.08	<.001	(0.77, 1.10)	
	Steatosis (LV)	-0.42	0.08	<.001	(-0.57, -0.27)	
	Inflammation (CD68)	0.04	0.10	.67	(-0.16, 0.24)	
T2*	Iron (Fe)	-0.34	0.08	<.001	(-0.51, -0.18)	0.60
	Fibrosis (CPA)	0.31	0.11	.005	(0.10, 0.53)	
	Steatosis (LV)	-0.12	0.10	.23	(-0.31, 0.08)	
	Inflammation (CD68)	0.54	0.13	<.001	(0.28, 0.80)	
T2	Iron (Fe)	0.00	0.03	.96	(-0.06, 0.06)	0.95
	Fibrosis	0.00	0.04	.90	(-0.07,	

MRI Metric	Predictor	β (Standardized)	Std. Error	P Value	95% CI	Adj. R^2
	(CPA)				0.08)	
	Steatosis (LV)	0.91	0.03	<.001	(0.84, 0.98)	
	Inflammation (CD68)	0.09	0.05	.05	(0.00, 0.19)	
PDFF	Iron (Fe)	-0.03	0.03	.24	(-0.08, 0.02)	0.96
	Fibrosis (CPA)	-0.16	0.03	<.001	(-0.23, -0.09)	
	Steatosis (LV)	1.00	0.03	<.001	(0.94, 1.06)	
	Inflammation (CD68)	-0.03	0.04	.53	(-0.11, 0.06)	

Note.—Standardized multivariable linear regression models were fit with each MRI metric (T1, FcT1, T2, T2*, PDFF) as the dependent variable and the four pathology metrics (Fe%, LV%, CPA%, CD68%) as simultaneous predictors; all continuous variables were z-standardized. Models in this table did not include experimental timepoint/group indicators. Timepoint-adjusted sensitivity analyses are provided in Supplementary Tables S14 and S15. β denotes the standardized regression coefficient (per 1 SD change in predictor). 95% confidence intervals, P values, and adjusted R^2 are reported for each full model.

Abbreviations: Adj. R^2 = adjusted coefficient of determination; β = standardized regression coefficient; CI = confidence interval; CD68 = CD68-positive area fraction; CPA = collagen proportionate area; Fe = hepatic iron area fraction on Perls' Prussian blue stain (Fe%); LV = lipid vacuole area fraction; FcT1 = fat-corrected T1; T1 = longitudinal relaxation time; T2 = transverse relaxation time; T2* = apparent transverse relaxation time; PDFF = proton density fat fraction.

Supplementary Table S3. Spearman Partial Correlations Between Quantitative MRI Metrics and Histopathological Metrics Adjusted for Hepatic Iron Deposition (Fe)

MRI Metric	Pathology	Spearman ρ	P Value
**FcT1**	Fibrosis (CPA)	0.24	.02
	Inflammation (CD68)	0.37	<.001
	Steatosis (LV)	0.34	.001
**T1**	Fibrosis (CPA)	0.32	.002
	Inflammation (CD68)	-0.02	.83
	Steatosis (LV)	-0.48	<.001
**T2**	Fibrosis (CPA)	0.12	.27
	Inflammation (CD68)	0.68	<.001
	Steatosis (LV)	0.90	<.001
**T2***	Fibrosis (CPA)	0.09	.40
	Inflammation (CD68)	0.64	<.001
	Steatosis (LV)	0.36	<.001
**PDFF**	Fibrosis (CPA)	-0.17	.11

MRI Metric	Pathology	Spearman ρ	P Value
	Inflammation (CD68)	0.48	<.001
	Steatosis (LV)	0.88	<.001

Note.— Spearman partial correlation coefficients (ρ) and corresponding P values are reported for each MRI–pathology pair after adjusting for hepatic iron deposition (Fe) as a covariate. Because iron deposition can influence relaxation measurements (e.g., T1/T2*), Fe was treated as a potential confounder and was included as an adjustment variable rather than a primary pathology endpoint. Accordingly, Fe is not displayed as a row/column variable in the correlation table (the table focuses on the associations between MRI metrics and LV/CPA/CD68 conditional on Fe). P values are from the partial-correlation test and are presented as nominal values (no multiple-comparison adjustment is applied in this table unless otherwise stated).

Abbreviations: CD68 = CD68-positive area fraction; CPA = collagen proportionate area; FcT1 = fat-corrected T1; Fe = Perls' Prussian blue–positive area fraction (%); LV = lipid vacuole area fraction; MRI = magnetic resonance imaging; P = P value; PDFF = proton density fat fraction; T1 = longitudinal relaxation time; T2 = transverse relaxation time; T2* = apparent transverse relaxation time; ρ = (partial) Spearman correlation coefficient.

Supplementary Table S4. Internal Validation of Diagnostic Performance Using Bootstrap Resampling with Out-of-Bag Evaluation

Target	Metric	AUC (95% CI)	Sensitivity (%)	Specificity (%)	Accuracy (%)
High Inflammation (CD68 > 2.33%)	T2	0.86 (0.75–0.95)	73.3 (50.0–93.3)	83.3 (55.0–100.0)	77.4 (65.5–88.2)
	T2*	0.80 (0.67–0.92)	64.7 (42.9–85.0)	90.0 (59.9–100.0)	77.1 (61.3–88.9)
	FcT1	0.68 (0.51–0.82)	45.5 (11.8–88.2)	68.8 (17.6–100.0)	57.6 (43.3–70.6)
	PDFF	0.79 (0.65–0.91)	85.0 (66.7–100.0)	68.8 (47.1–87.5)	76.6 (63.6–87.9)
Fibrosis (CPA > 1.95%)	T1	0.64 (0.25–0.83)	40.0 (10.0–66.7)	95.8 (59.1–100.0)	76.5 (52.8–88.2)
	FcT1	0.55 (0.23–0.74)	33.3 (7.1–57.1)	100.0 (55.0–100.0)	74.2 (45.5–86.1)
Fibrosis (CPA > 2.42%)	T1	0.77 (0.58–0.93)	50.0 (20.0–83.3)	96.0 (45.8–100.0)	81.5 (54.5–91.7)
	FcT1	0.57 (0.17–0.79)	40.0 (8.3–66.7)	100.0 (65.2–100.0)	80.0 (52.9–91.2)
Fibrosis (CPA > 2.90%)	T1	0.78 (0.60–0.94)	53.8 (22.2–87.5)	95.8 (50.0–100.0)	82.4 (57.6–93.3)
	FcT1	0.54 (0.19–0.76)	40.0 (7.1–66.7)	100.0 (63.6–100.0)	81.8 (51.5–93.1)

Note.—Internal validation was performed using bootstrap resampling at the animal level (B = 2000). For each replicate, the model and ROC cutoff were derived in the bootstrap sample

(training set) and performance was evaluated on the out-of-bag observations (test set). The cutoff was selected using the Youden index in the training set. AUC, sensitivity, specificity, and accuracy are summarized as the median (2.5th–97.5th percentiles) across replicates.

Abbreviations: AUC = area under the receiver operating characteristic curve; CI = confidence interval; FcT1 = fat-corrected T1; T1 = longitudinal relaxation time; T2 = transverse relaxation time; T2* = apparent transverse relaxation tim; PDFF = proton density fat fraction.

Supplementary Table S5. Internal Validation of Diagnostic Performance Using Repeated Stratified 10-Fold Cross-Validation

Target	Metric	AUC (95% CI)	Sensitivity (%)	Specificity (%)	Accuracy (%)
High Inflammation (CD68 > 2.33%)	T2	0.88 (0.51–1.00)	75.0 (25.0–100.0)	80.0 (40.0–100.0)	75.0 (50.0–100.0)
	T2*	0.81 (0.44–1.00)	75.0 (25.0–100.0)	100.0 (60.0–100.0)	80.0 (50.0–100.0)
	FcT1	0.69 (0.31–1.00)	40.0 (0.0–100.0)	60.0 (20.0–100.0)	50.0 (25.0–75.0)
	PDFF	0.81 (0.44–1.00)	80.0 (50.0–100.0)	75.0 (20.0–100.0)	80.0 (50.0–100.0)
Fibrosis (CPA > 1.95%)	T1	0.67 (0.19–1.00)	33.3 (0.0–100.0)	100.0 (80.0–100.0)	77.8 (60.0–100.0)
	FcT1	0.61 (0.12–1.00)	33.3 (0.0–100.0)	100.0 (80.0–100.0)	77.8 (55.6–100.0)
Fibrosis (CPA > 2.42%)	T1	0.81 (0.33–1.00)	50.0 (0.0–100.0)	100.0 (83.3–100.0)	87.5 (62.5–100.0)
	FcT1	0.67 (0.07–1.00)	33.3 (0.0–100.0)	100.0 (83.3–100.0)	80.0 (62.5–100.0)
Fibrosis (CPA > 2.90%)	T1	0.81 (0.33–1.00)	50.0 (0.0–100.0)	100.0 (83.3–100.0)	87.5 (62.5–100.0)
	FcT1	0.62	50.0 (0.0–	100.0	87.5 (66.7

Target	Metric	AUC (95% CI)	Sensitivity (%)	Specificity (%)	Accuracy (%)
		(0.02–1.00)	100.0)	(100.0–100.0)	–100.0)

Note.—Internal validation was also performed using repeated stratified 10-fold cross-validation (50 repeats; 500 test folds total). In each split, the ROC curve and Youden-optimal cutoff were derived in the training folds and evaluated in the held-out fold. AUC, sensitivity, specificity, and accuracy are summarized as the median (2.5th–97.5th percentiles) across test folds.

Abbreviations: AUC = area under the receiver operating characteristic curve; CI = confidence interval; FcT1 = fat-corrected T1; PDFF = proton density fat fraction; T1 = longitudinal relaxation time; T2 = transverse relaxation time; T2* = apparent transverse relaxation time.

Supplementary Table S6. Leave-One-Timepoint-Out Cross-Validation for Discriminating High Inflammation (CD68 > 2.33%): Fold-Level Results for T2 and T2*

Metric	Fold ID	AUC	Sensitivity	Specificity	Accuracy
T2	0	-	-	6/6 (100.0%)	6/6 (100.0%)
	2	0.62	0/2 (0.0%)	4/4 (100.0%)	4/6 (66.7%)
	4	0.38	0/4 (0.0%)	2/2 (100.0%)	2/6 (33.3%)
	7	0.70	4/4 (100.0%)	0/8 (0.0%)	4/12 (33.3%)
	14	0.28	8/8 (100.0%)	0/4 (0.0%)	8/12 (66.7%)
	28	-	12/12 (100.0%)	-	12/12 (100.0%)
	63	-	12/12 (100.0%)	-	12/12 (100.0%)
	F4N20	0.82	0/1 (0.0%)	11/11 (100.0%)	11/12 (91.7%)
	F9N15	0.75	0/2 (0.0%)	10/10 (100.0%)	10/12 (83.3%)
T2*	0	-	-	4/6 (66.7%)	4/6 (66.7%)
	2	1.00	2/2 (100.0%)	3/4 (75.0%)	5/6 (83.3%)
	4	0.12	0/4 (0.0%)	2/2 (100.0%)	2/6 (33.3%)
	7	0.97	4/4 (100.0%)	7/8 (87.5%)	11/12 (91.7%)
	14	0.88	6/8 (75.0%)	4/4 (100.0%)	10/12 (83.3%)
	28	-	6/12 (50.0%)	-	6/12 (50.0%)
	63	-	12/12 (100.0%)	-	12/12 (100.0%)
	F4N20	0.55	0/1 (0.0%)	11/11 (100.0%)	11/12 (91.7%)
	F9N15	0.85	0/2 (0.0%)	10/10 (100.0%)	10/12 (83.3%)

Note.— Leave-one-timepoint-out (LOGO) validation was performed by treating each experimental timepoint/group (Day0, 2, 4, 7, 14, 28, 63, F4N20, F9N15) as one held-out test group. Fold-level results are shown for T2 and T2* only; summary results for all metrics are provided in Supplementary Table S7.

In each split, the ROC curve was fit in the training set and the optimal cutoff was determined using the Youden index; the cutoff and ROC direction were then applied to the held-out group to compute sensitivity, specificity, accuracy, and confusion matrix counts. High inflammation was defined as CD68-positive area fraction > 2.33%. AUC in the test set was computed only when the held-out group contained both outcome classes. Folds with both outcome classes in the held-out group were considered valid folds (AUC computable); the number of valid folds is summarized as n/N in Supplementary Table S6.

Abbreviations: AUC = area under the receiver operating characteristic curve; T2 = transverse relaxation time; T2* = apparent transverse relaxation time.

Supplementary Table S7. Leave-One-Timepoint-Out Cross-Validation for Discriminating High Inflammation (CD68 > 2.33%): Summary Across Splits

Metric	Valid Folds (n/N)	Median AUC (95% CI)	Sensitivity	Specificity	Accuracy
T2	6/9	0.66 (0.29–0.81)	36/45 (80.0%)	33/45 (73.3%)	69/90 (76.7%)
T2*	6/9	0.86 (0.18–1.00)	30/45 (66.7%)	41/45 (91.1%)	71/90 (78.9%)
T1	6/9	0.42 (0.13–0.93)	17/45 (37.8%)	41/45 (91.1%)	58/90 (64.4%)
FcT1	6/9	0.57 (0.06–0.88)	15/45 (33.3%)	32/45 (71.1%)	47/90 (52.2%)
PDFF	6/9	0.41 (0.01–0.62)	40/45 (88.9%)	31/45 (68.9%)	71/90 (78.9%)

Note.— Data are numbers (numerator/denominator) with percentages in parentheses, or medians with 2.5th–97.5th percentiles in parentheses. Leave-one-timepoint-out cross-validation was performed by treating each experimental timepoint/group as one held-out test group (total N = 9 groups). Valid folds (n/N) indicates the number of held-out groups containing both outcome classes (AUC computable) divided by the total number of groups. High inflammation was defined as CD68-positive area fraction > 2.33%.

Abbreviations: AUC = area under the receiver operating characteristic curve; CI = confidence interval; CD68 = CD68-positive area fraction; FcT1 = fat-corrected

T1; T1 = longitudinal relaxation time; PDFF = proton density fat fraction; T2 = transverse relaxation time; T2* = apparent transverse relaxation time

Supplementary Table S8. Leave-One-Timepoint-Out Cross-Validation for Discriminating High Fibrosis (CPA > 1.95%, > 2.42%, and > 2.90%): Fold-Level Results

CPA Cutoff (%)	Metrics	Fold	AUC	Sensitivity	Specificity	Accuracy
1.95	FcT1	0	-	0/0 (-)	6/6 (100.0%)	6/6 (100.0%)
		14	-	0/0 (-)	12/12 (100.0%)	12/12 (100.0%)
		2	-	0/0 (-)	6/6 (100.0%)	6/6 (100.0%)
		28	0.69	0/8 (0.0%)	4/4 (100.0%)	4/12 (33.3%)
		4	-	0/0 (-)	6/6 (100.0%)	6/6 (100.0%)
		63	-	0/12 (0.0%)	0/0 (-)	0/12 (0.0%)
		7	-	0/0 (-)	10/12 (83.3%)	10/12 (83.3%)
		F4N20	-	0/0 (-)	12/12 (100.0%)	12/12 (100.0%)
		F9N15	-	0/12 (0.0%)	0/0 (-)	0/12 (0.0%)
	T1	0	-	0/0 (-)	6/6 (100.0%)	6/6 (100.0%)
		14	-	0/0 (-)	12/12 (100.0%)	12/12 (100.0%)
		2	-	0/0 (-)	6/6 (100.0%)	6/6 (100.0%)
		28	0.66	0/8 (0.0%)	4/4	4/12 (33.3%)

CPA Cutoff (%)	Metrics	Fold	AUC	Sensitivity	Specificity	Accuracy
					(100.0%)	
		4	-	0/0 (-)	6/6 (100.0%)	6/6 (100.0%)
		63	-	0/12 (0.0%)	0/0 (-)	0/12 (0.0%)
		7	-	0/0 (-)	12/12 (100.0%)	12/12 (100.0%)
		F4N20	-	0/0 (-)	10/12 (83.3%)	10/12 (83.3%)
		F9N15	-	0/12 (0.0%)	0/0 (-)	0/12 (0.0%)
2.42	FcT1	0	-	0/0 (-)	6/6 (100.0%)	6/6 (100.0%)
		14	-	0/0 (-)	12/12 (100.0%)	12/12 (100.0%)
		2	-	0/0 (-)	6/6 (100.0%)	6/6 (100.0%)
		28	0.56	0/3 (0.0%)	9/9 (100.0%)	9/12 (75.0%)
		4	-	0/0 (-)	6/6 (100.0%)	6/6 (100.0%)
		63	-	0/12 (0.0%)	0/0 (-)	0/12 (0.0%)
		7	-	0/0 (-)	10/12 (83.3%)	10/12 (83.3%)
		F4N20	-	0/0 (-)	12/12 (100.0%)	12/12 (100.0%)
		F9N15	-	0/12 (0.0%)	0/0 (-)	0/12 (0.0%)

CPA Cutoff (%)	Metrics	Fold	AUC	Sensitivity	Specificity	Accuracy
	T1	0	-	0/0 (-)	6/6 (100.0%)	6/6 (100.0%)
		14	-	0/0 (-)	12/12 (100.0%)	12/12 (100.0%)
		2	-	0/0 (-)	6/6 (100.0%)	6/6 (100.0%)
		28	0.63	0/3 (0.0%)	9/9 (100.0%)	9/12 (75.0%)
		4	-	0/0 (-)	6/6 (100.0%)	6/6 (100.0%)
		63	-	12/12 (100.0%)	0/0 (-)	12/12 (100.0%)
		7	-	0/0 (-)	12/12 (100.0%)	12/12 (100.0%)
		F4N20	-	0/0 (-)	10/12 (83.3%)	10/12 (83.3%)
		F9N15	-	0/12 (0.0%)	0/0 (-)	0/12 (0.0%)
2.90	FcT1	0	-	0/0 (-)	6/6 (100.0%)	6/6 (100.0%)
		14	-	0/0 (-)	12/12 (100.0%)	12/12 (100.0%)
		2	-	0/0 (-)	6/6 (100.0%)	6/6 (100.0%)
		28	0.30	0/2 (0.0%)	10/10 (100.0%)	10/12 (83.3%)
		4	-	0/0 (-)	6/6 (100.0%)	6/6 (100.0%)
		63	-	0/12 (0.0%)	0/0 (-)	0/12 (0.0%)

CPA Cutoff (%)	Metrics	Fold	AUC	Sensitivity	Specificity	Accuracy
		7	-	0/0 (-)	12/12 (100.0%)	12/12 (100.0%)
		F4N20	-	0/0 (-)	12/12 (100.0%)	12/12 (100.0%)
		F9N15	-	0/12 (0.0%)	0/0 (-)	0/12 (0.0%)
	T1	0	-	0/0 (-)	6/6 (100.0%)	6/6 (100.0%)
		14	-	0/0 (-)	12/12 (100.0%)	12/12 (100.0%)
		2	-	0/0 (-)	6/6 (100.0%)	6/6 (100.0%)
		28	0.40	0/2 (0.0%)	10/10 (100.0%)	10/12 (83.3%)
		4	-	0/0 (-)	6/6 (100.0%)	6/6 (100.0%)
		63	-	12/12 (100.0%)	0/0 (-)	12/12 (100.0%)
		7	-	0/0 (-)	12/12 (100.0%)	12/12 (100.0%)
		F4N20	-	0/0 (-)	10/12 (83.3%)	10/12 (83.3%)
		F9N15	-	0/12 (0.0%)	0/0 (-)	0/12 (0.0%)

Note.— Leave-one-timepoint-out (LOGO) validation was performed by withholding one experimental timepoint/group at a time as the test set (Day0, Day2, Day4, Day7, Day14, Day28, Day63, F4N20, and F9N15). In each split, the

ROC curve (including direction) and the Youden-optimal cutoff were determined in the training set and then applied unchanged to the held-out group to compute test performance. High fibrosis was defined as collagen proportionate area (CPA) exceeding 1.95%, 2.42%, or 2.90% (Target column). Test-set AUC was computed only when the held-out group contained both outcome classes. Sensitivity and specificity are reported as numerator/denominator (percentage) and are shown as "—" when undefined (e.g., no positive or no negative cases in the held-out group).

Abbreviations: AUC = area under the receiver operating characteristic curve; CPA = collagen proportionate area; FcT1 = fat-corrected T1; T1 = longitudinal relaxation time.

Supplementary Table S9. Leave-One-Timepoint-Out Cross-Validation for Discriminating High Fibrosis (CPA > 1.95%, > 2.42%, and > 2.90%): Summary Across Splits

Target	Metric	Valid Folds (n/N)	Median AUC (95% CI)	Sensitivity	Specificity	Accuracy
Fibrosis (CPA > 1.95%)	T1	1/9	0.66 (0.66–0.66)	0/32 (0.0%)	56/58 (96.6%)	56/90 (62.2%)
	FcT1	1/9	0.69 (0.69–0.69)	0/32 (0.0%)	56/58 (96.6%)	56/90 (62.2%)
Fibrosis (CPA > 2.42%)	T1	1/9	0.63 (0.63–0.63)	12/27 (44.4%)	61/63 (96.8%)	73/90 (81.1%)
	FcT1	1/9	0.56 (0.56–0.56)	0/27 (0.0%)	61/63 (96.8%)	61/90 (67.8%)
Fibrosis (CPA > 2.90%)	T1	1/9	0.40 (0.40–0.40)	12/26 (46.2%)	62/64 (96.9%)	74/90 (82.2%)
	FcT1	1/9	0.30 (0.30–0.30)	0/26 (0.0%)	64/64 (100.0%)	64/90 (71.1%)

Note.— Summary of leave-one-group-out cross-validation results across experimental timepoint/groups (N = 9) for each fibrosis definition (CPA > 1.95%, > 2.42%, > 2.90%). Valid folds (n/N) indicates the number of held-out groups containing both outcome classes (AUC computable) divided by the total number of groups. Median AUC is summarized across valid folds; the 95% interval corresponds to the 2.5th–97.5th percentiles across valid folds. Overall sensitivity,

specificity, and accuracy were computed from pooled confusion-matrix counts across all held-out predictions and are reported as numerator/denominator (percentage).

Abbreviations: CPA = collagen proportionate area; FcT1 = fat-corrected T1; T1 = longitudinal relaxation time.

Supplementary Table S10. GroupKFold Validation for Discriminating High Inflammation (CD68 > 2.33%): Fold-Level Results

Metric	Fold ID	AUC	Sensitivity	Specificity	Accuracy
T2	Fold1	0.97	12/13 (92.3%)	17/17 (100.0%)	29/30 (96.7%)
	Fold2	0.95	12/14 (85.7%)	4/4 (100.0%)	16/18 (88.9%)
	Fold3	0.68	12/18 (66.7%)	13/24 (54.2%)	25/42 (59.5%)
T2*	Fold1	0.66	4/13 (30.8%)	15/17 (88.2%)	19/30 (63.3%)
	Fold2	1.00	14/14 (100.0%)	3/4 (75.0%)	17/18 (94.4%)
	Fold3	0.78	10/18 (55.6%)	23/24 (95.8%)	33/42 (78.6%)
T1	Fold1	0.05	0/13 (0.0%)	17/17 (100.0%)	17/30 (56.7%)
	Fold2	0.02	0/14 (0.0%)	3/4 (75.0%)	3/18 (16.7%)
	Fold3	0.32	0/18 (0.0%)	24/24 (100.0%)	24/42 (57.1%)
FcT1	Fold1	0.59	0/13 (0.0%)	17/17 (100.0%)	17/30 (56.7%)
	Fold2	0.98	14/14 (100.0%)	1/4 (25.0%)	15/18 (83.3%)
	Fold3	0.49	3/18 (16.7%)	20/24 (83.3%)	23/42 (54.8%)
PDFF	Fold1	0.93	12/13 (92.3%)	17/17 (100.0%)	29/30 (96.7%)
	Fold2	0.91	12/14 (85.7%)	4/4 (100.0%)	16/18 (88.9%)
	Fold3	0.61	16/18 (88.9%)	10/24 (41.7%)	26/42 (61.9%)

Note.— GroupKFold cross-validation was performed using experimental timepoint/group as the grouping factor (k = 3), such that all observations from the same group were assigned to the same fold. In each split, the ROC curve and Youden-optimal cutoff were derived in the training folds and evaluated in the held-out fold. Sensitivity and specificity are reported as numerator/denominator (percentage). AUC is reported only when the held-out fold contained both outcome classes.

Abbreviations: AUC = area under the receiver operating characteristic curve; CD68 = CD68-positive area fraction; T2 = transverse relaxation time; T2* = apparent transverse relaxation time; FcT1 = fat-corrected T1; T1 = longitudinal relaxation time; PDFF = proton density fat fraction.

Supplementary Table S11. GroupKFold Validation for Discriminating High Inflammation (CD68 > 2.33%): Summary Across Folds

Metric	Valid Folds (n/N)	Median AUC (95% CI)	Sensitivity	Specificity	Accuracy
T2	3/3	0.95 (0.70–0.97)	36/45 (80.0%)	34/45 (75.6%)	70/90 (77.8%)
T2*	3/3	0.78 (0.67–0.99)	28/45 (62.2%)	41/45 (91.1%)	69/90 (76.7%)
T1	3/3	0.05 (0.02–0.31)	0/45 (0.0%)	44/45 (97.8%)	44/90 (48.9%)
FcT1	3/3	0.59 (0.49–0.96)	17/45 (37.8%)	38/45 (84.4%)	55/90 (61.1%)
PDFF	3/3	0.91 (0.63–0.92)	40/45 (88.9%)	31/45 (68.9%)	71/90 (78.9%)

Note.— Summary of GroupKFold results across folds (k = 3). Valid folds (n/N) indicates the number of held-out folds containing both outcome classes (AUC computable) divided by the total number of folds. Median AUC is summarized across valid folds; the 95% interval corresponds to the 2.5th–97.5th percentiles across valid folds. Overall sensitivity, specificity, and accuracy were computed from pooled confusion-matrix counts across all held-out predictions and are reported as numerator/denominator (percentage).

Abbreviations: AUC = area under the receiver operating characteristic curve; CD68 = CD68-positive area fraction; T2 = transverse relaxation time; T2* =

apparent transverse relaxation time; FcT1 = fat-corrected T1; T1 = longitudinal relaxation time; PDFF = proton density fat fraction.

Supplementary Table S12. GroupKFold Validation for Discriminating High Fibrosis (CPA > 1.95%, > 2.42%, and > 2.90%): Fold-Level Results

Target	Metric	Fold ID	AUC	Sensitivity	Specificity	Accuracy
Fibrosis (CPA > 1.95%)	T1	Fold1	-	-	-	-
		Fold2	1.00	12/12 (100.0%)	6/6 (100.0%)	18/18 (100.0%)
		Fold3	0.81	0/12 (0.0%)	30/30 (100.0%)	30/42 (71.4%)
Fibrosis (CPA > 2.42%)		Fold1	-	-	-	-
		Fold2	1.00	12/12 (100.0%)	0/6 (0.0%)	12/18 (66.7%)
		Fold3	0.81	0/12 (0.0%)	30/30 (100.0%)	30/42 (71.4%)
Fibrosis (CPA > 2.90%)		Fold1	-	-	-	-
		Fold2	1.00	12/12 (100.0%)	1/6 (16.7%)	13/18 (72.2%)
		Fold3	0.81	0/12 (0.0%)	30/30 (100.0%)	30/42 (71.4%)
Fibrosis (CPA > 1.95%)	FcT1	Fold1	0.68	0/8 (0.0%)	22/22 (100.0%)	22/30 (73.3%)
		Fold2	0.00	0/12 (0.0%)	3/6 (50.0%)	3/18 (16.7%)
		Fold3	0.16	0/12 (0.0%)	23/30 (76.7%)	23/42 (54.8%)

Target	Metric	Fold ID	AUC	Sensitivity	Specificity	Accuracy
Fibrosis (CPA > 2.42%)		Fold1	0.64	0/3 (0.0%)	27/27 (100.0%)	27/30 (90.0%)
		Fold2	0.00	0/12 (0.0%)	5/6 (83.3%)	5/18 (27.8%)
		Fold3	0.16	0/12 (0.0%)	23/30 (76.7%)	23/42 (54.8%)
Fibrosis (CPA > 2.90%)		Fold1	0.45	0/2 (0.0%)	28/28 (100.0%)	28/30 (93.3%)
		Fold2	0.00	0/12 (0.0%)	5/6 (83.3%)	5/18 (27.8%)
		Fold3	0.16	0/12 (0.0%)	30/30 (100.0%)	30/42 (71.4%)

Note.— Group k-fold cross-validation was performed using timepoint/groups as the grouping unit. For each split, the Youden-optimal cutoff was determined in the training set and evaluated in the held-out group fold. High fibrosis was defined as CPA > 1.95%, > 2.42%, and > 2.90%.

Abbreviations: AUC = area under the receiver operating characteristic curve; CPA = collagen proportionate area; FcT1 = fat-corrected T1; T1 = longitudinal relaxation time.

Supplementary Table S13. GroupKFold Validation for Discriminating High Fibrosis (CPA > 1.95%, > 2.42%, and > 2.90%): Summary Across Folds

Target	Metric	Valid Folds (n/N)	Median AUC (95% CI)	Sensitivity	Specificity	Accuracy
Fibrosis (CPA > 1.95%)	T1	2/3	0.91 (0.82–1.00)	12/24 (50.0%)	36/36 (100.0%)	48/60 (80.0%)
	FcT1	3/3	0.16 (0.01–0.65)	0/32 (0.0%)	48/58 (82.8%)	48/90 (53.3%)
Fibrosis (CPA > 2.42%)	T1	2/3	0.91 (0.82–1.00)	12/24 (50.0%)	30/36 (83.3%)	42/60 (70.0%)
	FcT1	3/3	0.16 (0.01–0.62)	0/27 (0.0%)	55/63 (87.3%)	55/90 (61.1%)
Fibrosis (CPA > 2.90%)	T1	2/3	0.91 (0.82–1.00)	12/24 (50.0%)	31/36 (86.1%)	43/60 (71.7%)
	FcT1	3/3	0.16 (0.01–0.43)	0/26 (0.0%)	63/64 (98.4%)	63/90 (70.0%)

Note.— Summary of GroupKFold results across folds (k = 3) for each fibrosis definition (CPA > 1.95%, > 2.42%, > 2.90%). Valid folds (n/N) indicates the number of held-out folds containing both outcome classes (AUC computable) divided by the total number of folds. Median AUC is summarized across valid folds; the 95% interval corresponds to the 2.5th–97.5th percentiles across valid folds. Overall sensitivity, specificity, and accuracy were computed from pooled

confusion-matrix counts across all held-out predictions and are reported as numerator/denominator (percentage).

Abbreviations: AUC = area under the receiver operating characteristic curve; CI = confidence interval; CPA = collagen proportionate area; FcT1 = fat-corrected T1; T1 = longitudinal relaxation time.

Supplementary Table S14. Spearman Partial Correlations Between MRI Metrics and Pathology Metrics Adjusted for Hepatic Iron and Timepoint

Metric	Pathology	Partial Rho	P Value	q Value (FDR)
T1	Steatosis (LV)	-0.40	<.001	.002
	Fibrosis (CPA)	-0.00	>.99	>.99
	Inflammation (CD68)	-0.15	.19	.34
FcT1	Steatosis (LV)	-0.25	.02	.06
	Fibrosis (CPA)	-0.04	.73	.87
	Inflammation (CD68)	-0.14	.20	.34
T2	Steatosis (LV)	0.34	.002	.009
	Fibrosis (CPA)	0.24	.03	.07
	Inflammation (CD68)	0.07	.56	.76
T2*	Steatosis (LV)	-0.04	.75	.87
	Fibrosis (CPA)	-0.09	.40	.60
	Inflammation (CD68)	0.46	<.001	<.001
PDFF	Steatosis (LV)	0.32	.003	.01
	Fibrosis (CPA)	0.02	.84	.90
	Inflammation (CD68)	-0.29	.008	.02

Note.— Partial Spearman correlation coefficients (ρ) were computed between MRI metrics (T1, FcT1, T2, T2*, PDFF) and pathology metrics (steatosis [LV%], fibrosis [CPA%], inflammation [CD68%]) while adjusting for hepatic iron deposition (Fe%, Perls stain–based hepatic iron area fraction) and experimental timepoint/group (modeled as categorical indicator variables). P values are from tests of the null hypothesis ρ = 0 for the partial correlation. q values are

Benjamini–Hochberg false discovery rate (BH–FDR)–adjusted P values across all MRI–pathology comparisons in this table.

Abbreviations: BH = Benjamini–Hochberg; ρ = Spearman partial correlation coefficient; FDR = false discovery rate; FcT1 = fat-corrected T1; PDFF = proton density fat fraction; T1 = longitudinal relaxation time; T2 = transverse relaxation time; T2* = apparent transverse relaxation time; Fe = hepatic iron area fraction on Perls' Prussian blue stain (Fe%); LV = lipid vacuole area fraction; CPA = collagen proportionate area; CD68 = CD68-positive area fraction.

Supplementary Table S15. Standardized Multivariable Regression Coefficients for Pathology Determinants of MRI Metrics Adjusted for Timepoint

Outcome	Predictor	Estimate (95% CI)	P Value	Adj R^2
T1	Fibrosis (CPA)	-0.09 (-0.40–0.23)	.60	0.90
	Steatosis (LV)	-0.50 (-0.90–-0.10)	.01	0.90
	Inflammation (CD68)	-0.07 (-0.23–0.09)	.41	0.90
	Iron (Fe)	-0.03 (-0.15–0.10)	.69	0.90
FcT1	Fibrosis (CPA)	-0.25 (-0.55–0.06)	.11	0.91
	Steatosis (LV)	-0.25 (-0.64–0.13)	.20	0.91
	Inflammation (CD68)	-0.10 (-0.26–0.05)	.20	0.91
	Iron (Fe)	-0.00 (-0.13–0.12)	.96	0.91
T2	Fibrosis (CPA)	-0.04 (-0.24–0.15)	.66	0.96
	Steatosis (LV)	0.67 (0.43–0.92)	<.001	0.96
	Inflammation (CD68)	-0.06 (-0.16–0.04)	.23	0.96
	Iron (Fe)	0.07 (-0.01–0.14)	.10	0.96
T2*	Fibrosis (CPA)	-0.11 (-0.67–0.44)	.69	0.70
	Steatosis (LV)	-0.62 (-1.32–0.08)	.08	0.70
	Inflammation (CD68)	0.62 (0.34–0.90)	<.001	0.70
	Iron (Fe)	-0.01 (-0.23–0.21)	.93	0.70
PDFF	Fibrosis (CPA)	-0.18 (-0.28–-0.07)	.001	0.99
	Steatosis (LV)	0.32 (0.19–0.45)	<.001	0.99
	Inflammation (CD68)	-0.06 (-0.11–-0.01)	.03	0.99
	Iron (Fe)	0.01 (-0.04–0.05)	.80	0.99

Note.— Standardized multivariable linear regression models were fit with each MRI metric as the dependent variable and pathology metrics (CPA, LV, CD68, Fe) as predictors, additionally adjusting for experimental timepoint/group (categorical). The table reports standardized beta coefficients (β), 95% confidence intervals, P values, and adjusted R^2 for the main pathology predictors.

(Timepoint-term coefficients are listed separately in Supplementary Table S16.)

Abbreviations: Adj.R^2 = adjusted coefficient of determination; β = standardized regression coefficient; CI = confidence interval; CD68 = CD68-positive area fraction; CPA = collagen proportionate area; Fe = hepatic iron area fraction on Perls' Prussian blue stain (Fe%); LV = lipid vacuole area fraction; FcT1 = fat-corrected T1; PDFF = proton density fat fraction; T1 = longitudinal relaxation time; T2 = transverse relaxation time; T2* = apparent transverse relaxation time.

Supplementary Table S16. Timepoint (Group) Terms From Standardized Multivariable Regression Models

Outcome	Timepoint	β estimate (95% CI)	P Value
T1	Day 2	-0.08 (-0.44–0.28)	.65
	Day 4	-0.11 (-0.53–0.31)	.60
	Day 7	0.11 (-0.59–0.81)	.76
	Day 14	0.24 (-0.68–1.17)	.60
	Day 28	0.06 (-1.00–1.11)	.91
	Day 63	3.38 (2.08–4.69)	<.001
	F4N20	-0.13 (-0.49–0.22)	.46
	F9N15	-0.01 (-0.63–0.61)	.96
FcT1	Day 2	-0.05 (-0.40–0.29)	.76
	Day 4	0.26 (-0.14–0.66)	.20
	Day 7	0.67 (-0.00–1.34)	.05
	Day 14	0.89 (-0.00–1.78)	.05
	Day 28	0.83 (-0.18–1.85)	.11
	Day 63	4.22 (2.96–5.47)	<.001
	F4N20	-0.14 (-0.48–0.21)	.44
	F9N15	0.12 (-0.47–0.72)	.68
T2	Day 2	-0.01 (-0.24–0.21)	.92
	Day 4	-0.13 (-0.39–0.13)	.32
	Day 7	0.03 (-0.41–0.47)	.89
	Day 14	0.49 (-0.09–1.06)	.10
	Day 28	0.70 (0.05–1.36)	.04

Outcome	Timepoint	β estimate (95% CI)	P Value
	Day 63	0.80 (-0.01–1.62)	.05
	F4N20	-0.23 (-0.45–-0.01)	.05
	F9N15	-0.35 (-0.73–0.04)	.08
T2*	Day 2	0.22 (-0.41–0.85)	.50
	Day 4	-0.41 (-1.14–0.33)	.27
	Day 7	1.05 (-0.18–2.27)	.09
	Day 14	0.79 (-0.84–2.41)	.34
	Day 28	0.64 (-1.21–2.48)	.49
	Day 63	1.92 (-0.36–4.21)	.10
	F4N20	-0.36 (-0.99–0.27)	.25
	F9N15	-0.60 (-1.69–0.48)	.27
PDFF	Day 2	0.02 (-0.10–0.14)	.75
	Day 4	0.79 (0.65–0.93)	<.001
	Day 7	1.25 (1.02–1.48)	<.001
	Day 14	1.44 (1.14–1.75)	<.001
	Day 28	1.72 (1.37–2.07)	<.001
	Day 63	1.54 (1.10–1.97)	<.001
	F4N20	-0.12 (-0.24–-0.00)	.05
	F9N15	0.04 (-0.16–0.25)	.67

Note.— This table lists the regression coefficients for experimental timepoint/group indicator terms from the standardized multivariable models described in Supplementary Table S14. F4N20 indicates choline-deficient, L-amino acid-defined, high-fat diet (CDAHFD) feeding for 4 weeks followed by

normal chow for 20 weeks, and F9N15 indicates CDAHFD feeding for 9 weeks followed by normal chow for 15 weeks. The reference level is Day 0. Each coefficient represents the standardized difference in the MRI outcome for the given timepoint/group relative to Day 0, after adjusting for pathology covariates.

Abbreviations: β = standardized regression coefficient; CI = confidence interval; FcT1 = fat-corrected T1; PDFF = proton density fat fraction; T1 = longitudinal relaxation time; T2 = transverse relaxation time; T2* = apparent transverse relaxation time.

Supplementary Table S17. Variance inflation factors for pathology predictors

Model	Predictor	R^2 for predictor model	VIF
Pathology-only model	Iron (Fe)	0.347	1.53
Pathology-only model	Steatosis (LV)	0.513	2.05
Pathology-only model	Fibrosis (CPA)	0.617	2.61
Pathology-only model	Inflammation (CD68)	0.733	3.74
Pathology + timepoint/group indicators	Iron (Fe)	0.731	3.72
Pathology + timepoint/group indicators	Steatosis (LV)	0.973	37.19
Pathology + timepoint/group indicators	Fibrosis (CPA)	0.958	23.58
Pathology + timepoint/group indicators	Inflammation (CD68)	0.833	5.99

Note.— Variance inflation factors (VIFs) were calculated for the pathology predictors used in the standardized multivariable regression models. The pathology-only model corresponds to the Figure 5B models. The timepoint/group-adjusted model additionally included experimental timepoint/group indicator variables. VIF = $1/(1 - R^2)$, where R^2 was obtained by regressing each pathology predictor on the remaining pathology predictors, with or without timepoint/group indicators. Pathology-only VIFs were below 5, supporting acceptable collinearity for the Figure 5B models. Higher VIFs after timepoint/group adjustment reflect co-evolution of steatosis and fibrosis with disease stage; these models were therefore interpreted as conservative sensitivity analyses. Abbreviations: CD68 = CD68-positive area fraction; CPA = collagen proportionate area; Fe = Perls' Prussian blue-positive area fraction; LV = lipid vacuole area fraction; VIF = variance inflation factor.

Reference

1. Lee J, Jung M, Park J, et al. Highly accelerated knee magnetic resonance imaging using deep neural network (DNN)-based reconstruction: prospective, multi-reader, multi-vendor study. Sci Rep 2023;13(1):17264. doi: 10.1038/s41598-023-44248-7
2. Yoo H, Yoo RE, Choi SH, et al. Deep learning-based reconstruction for acceleration of lumbar spine MRI: a prospective comparison with standard MRI. Eur Radiol 2023;33(12):8656-8668. doi: 10.1007/s00330-023-09918-0
3. Crooks LE, Arakawa M, Hoenninger J, McCarten B, Watts J, Kaufman L. Magnetic resonance imaging: effects of magnetic field strength. Radiology 1984;151(1):127-133. doi: 10.1148/radiology.151.1.6701302

Supplementary Appendix P: Technical validation (Figs. P1–P9; Tables P1–P7)

Overview

This Supplementary Appendix reports exploratory technical validation experiments performed to characterize measurement properties of the low-field T1 mapping protocol and reconstruction pipeline, not to establish quantitative superiority or clinical benefit of AIRS. Phantom experiments evaluated (i) cross-calibration between 2ptIR and multiTR T1 mapping, (ii) sensitivity of 2ptIR T1 to small flip-angle offsets, and (iii) the effect of AIRS reconstruction on phantom T1 (Figs. P1–P4; Tables P1–P3). In vivo experiments in normal rats evaluated test–retest repeatability and AIRS ON–OFF differences for both 2ptIR and multiTR (Figs. P5–P9; Tables P4–P7).

Common definitions (apply to all Figures and Tables).

AIRS = deep-learning–based reconstruction applied in the image domain to standard-reconstructed DICOM images (SwiftMR; AIRS Medical) (ON: applied; OFF: not applied).

2ptIR = two-point inversion recovery T1 mapping; multiTR = multiTR T1 mapping.

All T1 values are reported in milliseconds (ms). In vivo ROIs were placed in liver parenchyma while avoiding large vessels.

Methods

P.1 Phantom experiments

Design and acquisition.

A T1 phantom with multiple vials spanning the relevant T1 range was scanned on the 0.4-T open MRI system using the same 2ptIR and multiTR mapping protocols as described in the Supplementary Methods. For each acquisition, images were reconstructed with AIRS ON and AIRS OFF. Vial-wise mean T1 values were extracted for each session.

Cross-calibration (2ptIR vs multiTR).

Agreement between 2ptIR and multiTR was assessed per vial × session pair ($n = 24$) using ordinary least squares (OLS) regression and Bland–Altman analysis (Figs. P1–P2; Table P1). An a priori outlier rule was applied (|studentized residual| > 3 or Cook's distance > 4/n), and sensitivity analyses were repeated after excluding flagged points.

Flip-angle sensitivity.

Sensitivity of 2ptIR-derived T1 to a small flip-angle deviation was evaluated by repeating acquisitions with a +1° flip-angle perturbation; relative changes were summarized overall and by AIRS status (Fig. P3; Table P2).

Effect of AIRS reconstruction.

The effect of AIRS on measured T1 values was assessed within matched vial × session × method pairs (n = 24), including overall and method-specific analyses (Fig. P4; Table P3).

P.2 Normal rat experiments

Design and acquisition.

Normal rats were scanned on the same 0.4-T system. For each animal × session pair (n = 20), T1 maps were reconstructed with AIRS ON and AIRS OFF for both 2ptIR and multiTR. ROI placement followed the common rule (liver parenchyma avoiding vessels).

In vivo AIRS ON–OFF comparisons.

AIRS ON versus OFF values were compared using Bland–Altman analysis and OLS regression (Figs. P5–P8; Table P5). Raw paired values used for scatterplots are provided for transparency (Tables P6–P7).

Test–retest repeatability.

Test–retest repeatability was summarized for 2ptIR and multiTR with AIRS OFF and ON (n = 10 pairs per configuration) using Bland–Altman analysis and repeatability metrics; Figure P9 displays the 2ptIR AIRS OFF comparison (Table P4).

P.3 Statistical analysis (phantom and in vivo validation)

Regression.

OLS models were fit as: y = intercept + slope × x. For phantom cross-calibration, multiTR was modeled as a function of 2ptIR (multiTR ~ 2ptIR). For AIRS ON–OFF comparisons, T1_ON was modeled as a function of T1_OFF (T1_ON ~ T1_OFF).

Agreement.

Bland–Altman analysis reported bias (mean difference) and 95% limits of agreement (LoA = bias ± 1.96 × SD of differences). For test–retest, difference was defined as rep2 − rep1.

Repeatability metrics.

Within-subject SD (wSD) was computed as SD(diff)/sqrt(2), repeatability coefficient as RC95 = 1.96 × SD(diff), and within-subject coefficient of variation as wCV = 100 × wSD / mean(pair mean). ICC(A,1) (two-way mixed, absolute agreement, single measure) and Lin’s concordance correlation coefficient (CCC) were also reported (Table P4).

Uncertainty.

Uncertainty was summarized using 95% confidence intervals where applicable (Tables P1–P5). These technical analyses were exploratory and descriptive; no formal superiority or inferiority test between AIRS ON and OFF was prespecified.

Supplementary Figures (Appendix P)

Supplementary Figure P1. Cross-calibration: 2ptIR vs multiTR (phantom; per vial × session, n = 24).

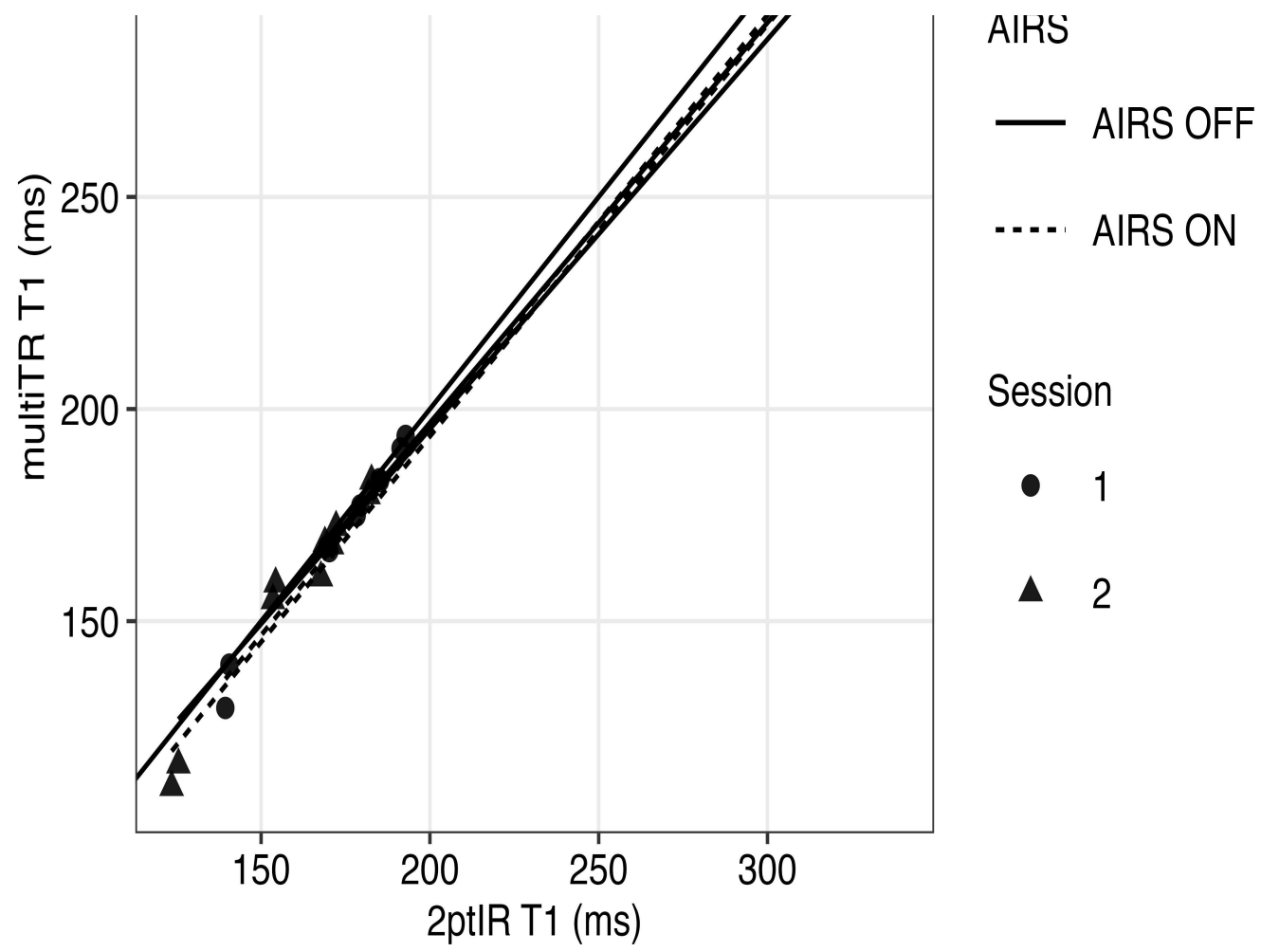

Scatterplot of vial × session mean T1 values (2ptIR vs multiTR). The black line indicates the identity (y = x). Separate OLS fits are shown for AIRS OFF and AIRS ON. Marker shape indicates session. **Note.**—Overall: slope 0.952 (95% CI: 0.922, 1.002), intercept 4.87 ms (95% CI: −4.49, 11.80), R^2 = 0.995, RMSE = 4.56 ms, MAPE = 2.73%. Bland–Altman bias −4.46 ms (95% CI: −6.80, −2.38); 95% LoA −15.56 ms to +6.63 ms. Outlier flagging: 4/24 points met the a priori rule (|studentized residual| > 3 or Cook’s D > 4/n); conclusions were unchanged in sensitivity analyses. **Abbreviations**: 2ptIR = two-point inversion recovery; AIRS = AI-based reconstruction software; CI = confidence interval; LoA = limits of agreement; MAPE = mean absolute percentage error; multiTR = multi-TR saturation recovery; OLS = ordinary least squares; RMSE = root mean square error.

Supplementary Figure P2. Bland–Altman: 2ptIR vs multiTR (phantom; n = 24).

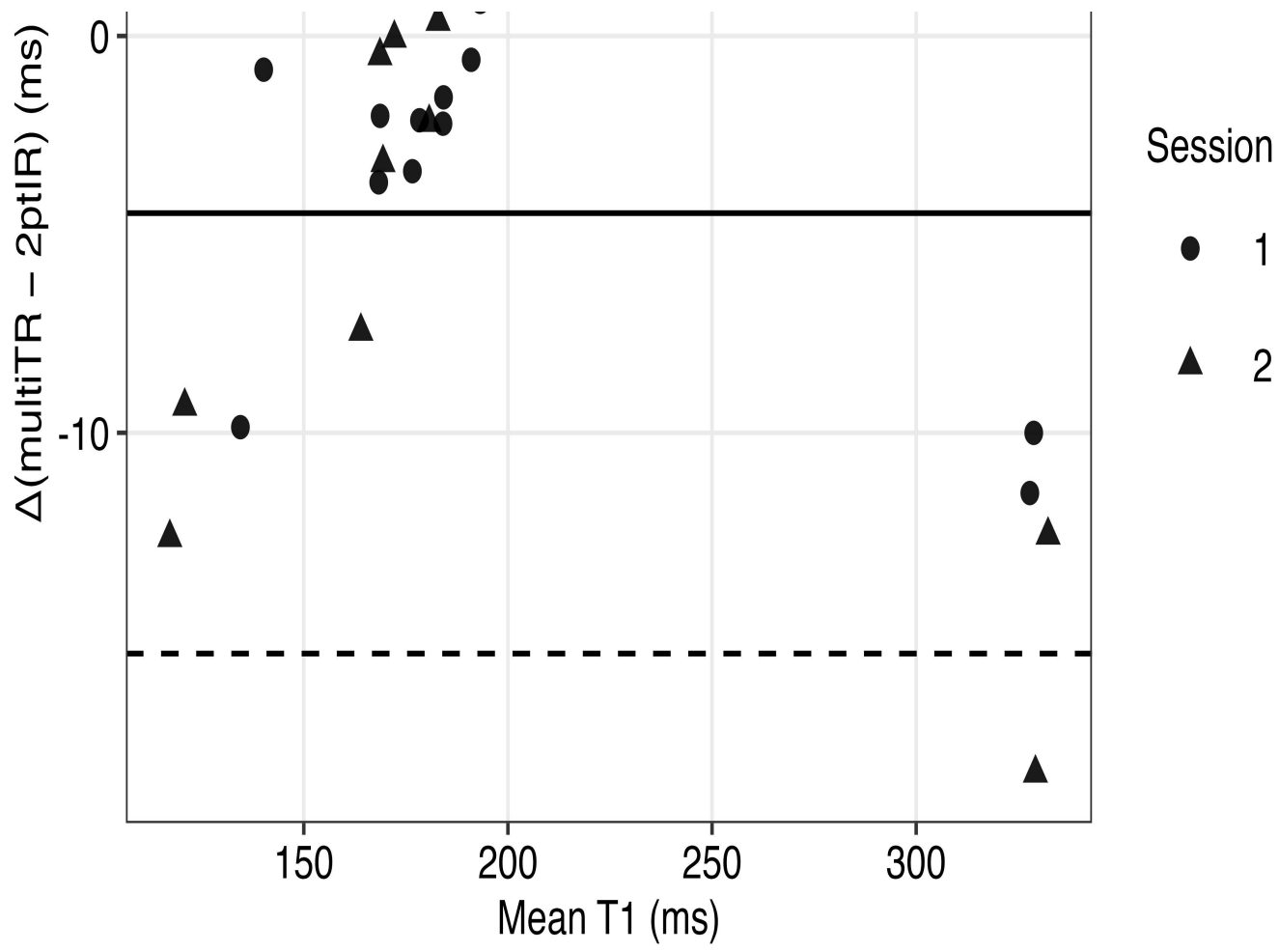

Bland–Altman plot comparing 2ptIR and multiTR T1 values. The solid line indicates the bias; dashed lines indicate the 95% LoA. Marker shape indicates session. **Note**.—Bias −4.46 ms (95% CI: −6.80, −2.38); 95% LoA −15.56 ms to +6.63 ms. **Abbreviations**: 2ptIR = two-point inversion recovery; CI = confidence interval; LoA = limits of agreement; multiTR = multi-TR saturation recovery.

Supplementary Figure P3. Flip-angle sensitivity of 2ptIR T1 (phantom; 0° baseline vs +1°) with distributional summaries.

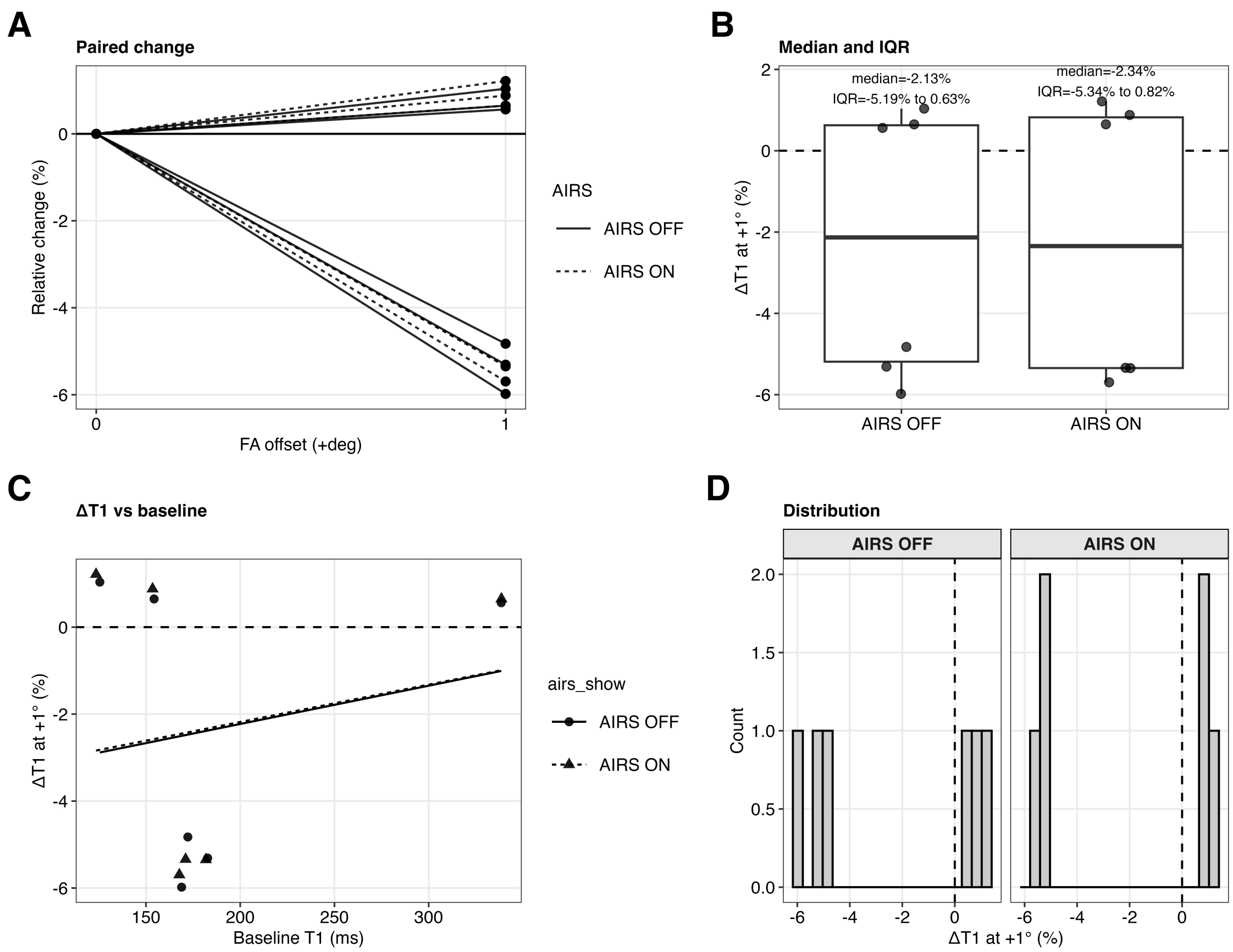

Relative percent change in 2ptIR T1 after a +1° flip-angle perturbation, expressed as the percent difference from baseline (2ptIR, 0°). **(A)** Individual vial × session trajectories connecting baseline (0°) and +1°; line type indicates AIRS status (OFF/ON). **(B)** Boxplots of ΔT1 at +1° stratified by AIRS, highlighting the median (center line) and interquartile range (IQR; box); points represent individual vial × session pairs. **(C)** Scatter plot of ΔT1 at +1° versus baseline T1 (0°) to assess whether flip-angle sensitivity depends on baseline T1 (i.e., whether the direction/magnitude differs for shorter vs longer baseline T1). **(D)** Histograms

of ΔT1 at +1° (faceted by AIRS) to visualize the distribution of increases and decreases; the vertical reference line indicates 0% change. **Note**.—+1° overall (n = 12): median −2.61% (95% CI: −5.90%, +0.60%), IQR −5.85% to +0.58%. By AIRS: OFF median −2.13% (IQR −5.19% to +0.63%); ON median −3.11% (IQR −5.95% to +0.17%). No outliers were flagged (MAD > 3 rule). **Abbreviations**: 2ptIR = two-point inversion recovery; AIRS = AI-based reconstruction software; CI = confidence interval; IQR = interquartile range; MAD = median absolute deviation.

Supplementary Figure P4. Effect of AIRS reconstruction on T1 (phantom; paired within vial × session × method, n = 24).

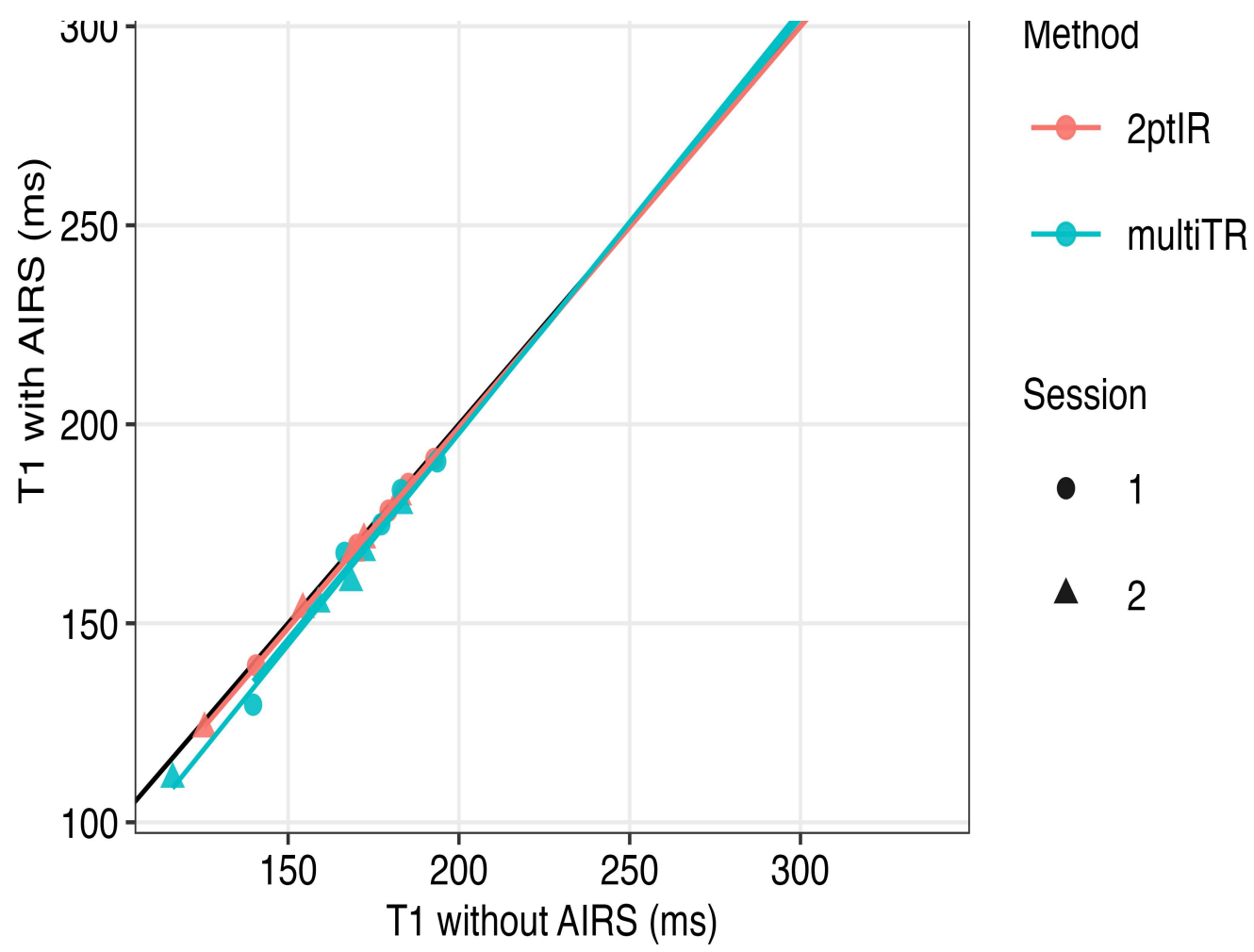

Scatterplot comparing AIRS ON (y-axis) vs AIRS OFF (x-axis). The identity line (y = x) is shown. Method-specific OLS fits are displayed. **Note.**—Overall: slope 1.029 (95% CI: 1.011, 1.047), intercept −7.33 ms (95% CI: −11.02, −3.64), R^2 = 0.998, RMSE = 2.60 ms, mean difference −1.73 ms (95% CI: −3.10, −0.36). 2ptIR: slope 1.008 (95% CI: 1.003, 1.012), intercept −2.32 ms (95% CI: −3.19, −1.46), RMSE 0.39 ms, mean difference −0.86 ms (95% CI: −1.27, −0.44). multiTR: slope 1.053 (95% CI: 1.020, 1.085), intercept −12.72 ms (95% CI: −19.24, −6.21), R^2 = 0.998, RMSE 2.81 ms, mean difference −2.61 ms (95% CI: −5.44, +0.23). **Abbreviations**: 2ptIR = two-point inversion recovery; AIRS = AI-based reconstruction software; CI = confidence interval; multiTR = multi-TR saturation recovery; OLS = ordinary least squares; R^2 = coefficient of determination; RMSE = root mean square error.

Supplementary Figure P5. Bland–Altman (AIRS ON − OFF): 2ptIR (normal rat liver; n = 20).

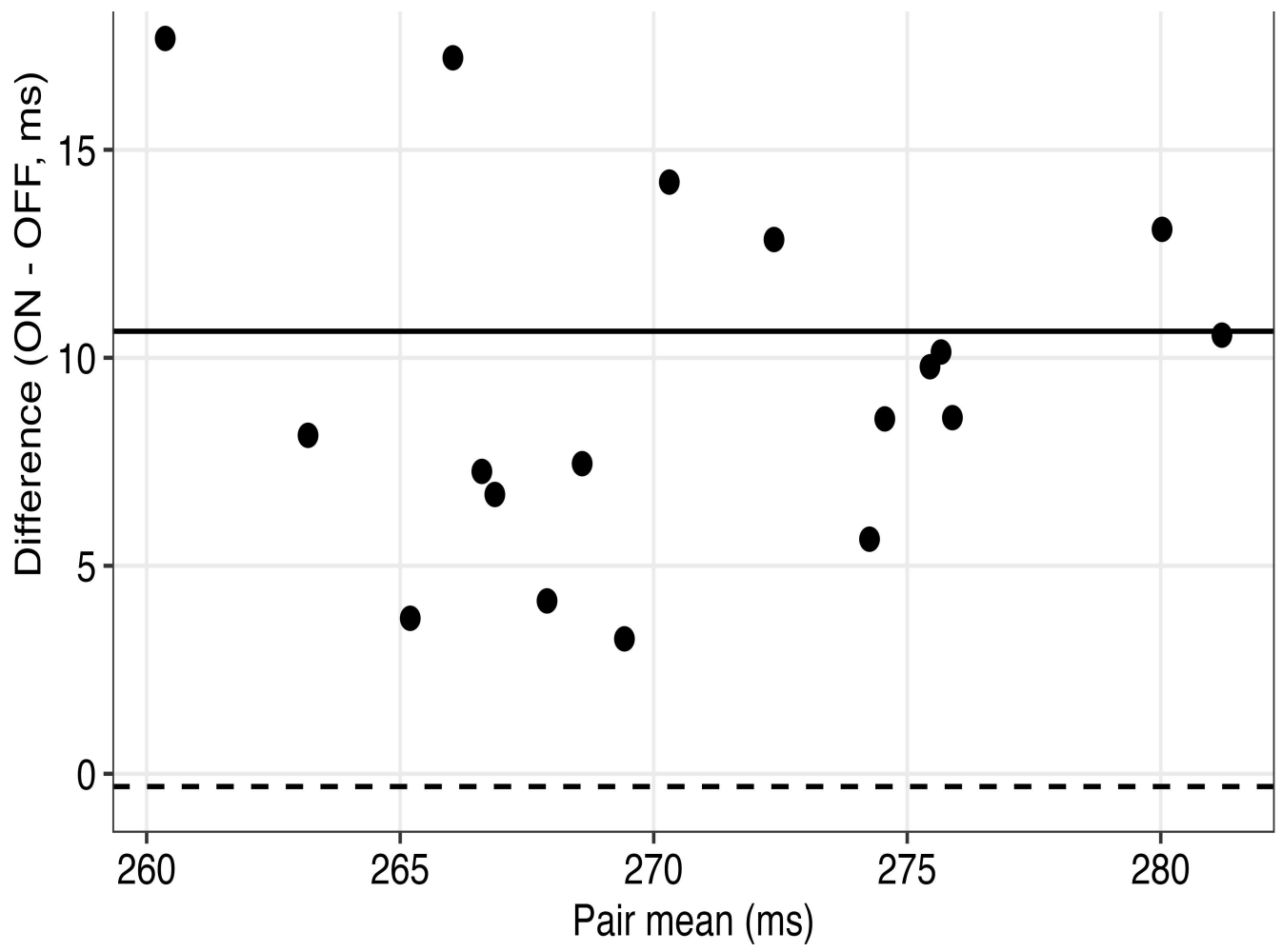

Bland–Altman plot comparing T1 values reconstructed with AIRS (ON) versus without AIRS (OFF) for 2ptIR. The vertical axis is the difference (ON − OFF, ms) and the horizontal axis is the pair mean (ms). Solid line indicates bias; dashed lines indicate 95% LoA. **Note**.—Positive differences indicate longer T1 with AIRS ON. Numerical summaries and regression estimates are provided in Table P5. **Abbreviations**: 2ptIR = two-point inversion recovery; AIRS = AI-based reconstruction software; LoA = limits of agreement.

Supplementary Figure P6. Bland–Altman (AIRS ON − OFF): multiTR (normal rat liver; n = 20).

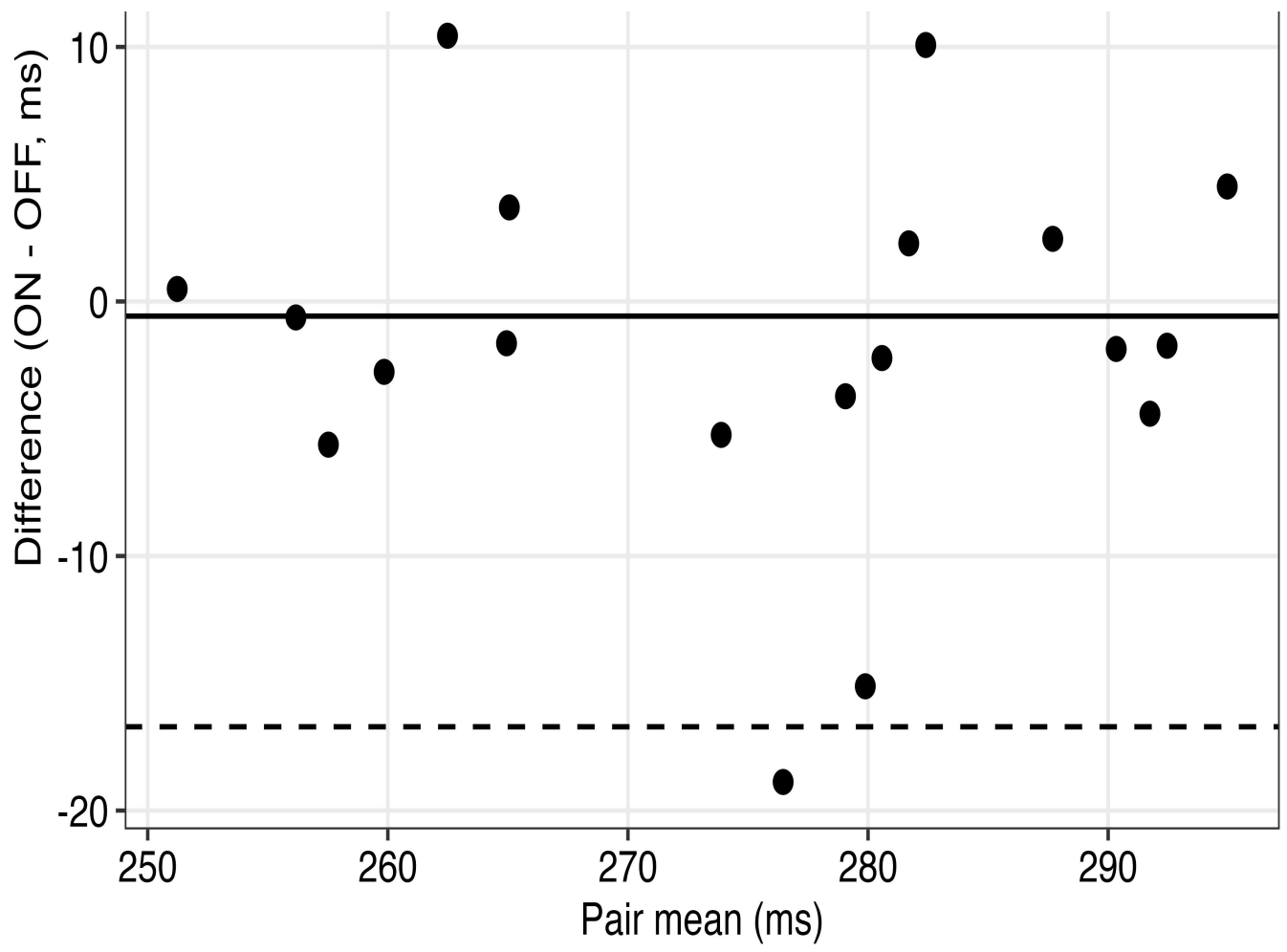

Bland–Altman plot for multiTR-based T1 mapping under AIRS ON vs OFF. Axis definitions and lines are as in Fig. P5. **Note**.—Numerical summaries and regression estimates are provided in Table P5. **Abbreviations**: AIRS = AI-based reconstruction software; LoA = limits of agreement; multiTR = multi-TR saturation recovery.

Supplementary Figure P7. AIRS ON vs OFF (2ptIR): identity line and OLS fit (normal rat liver; n = 20).

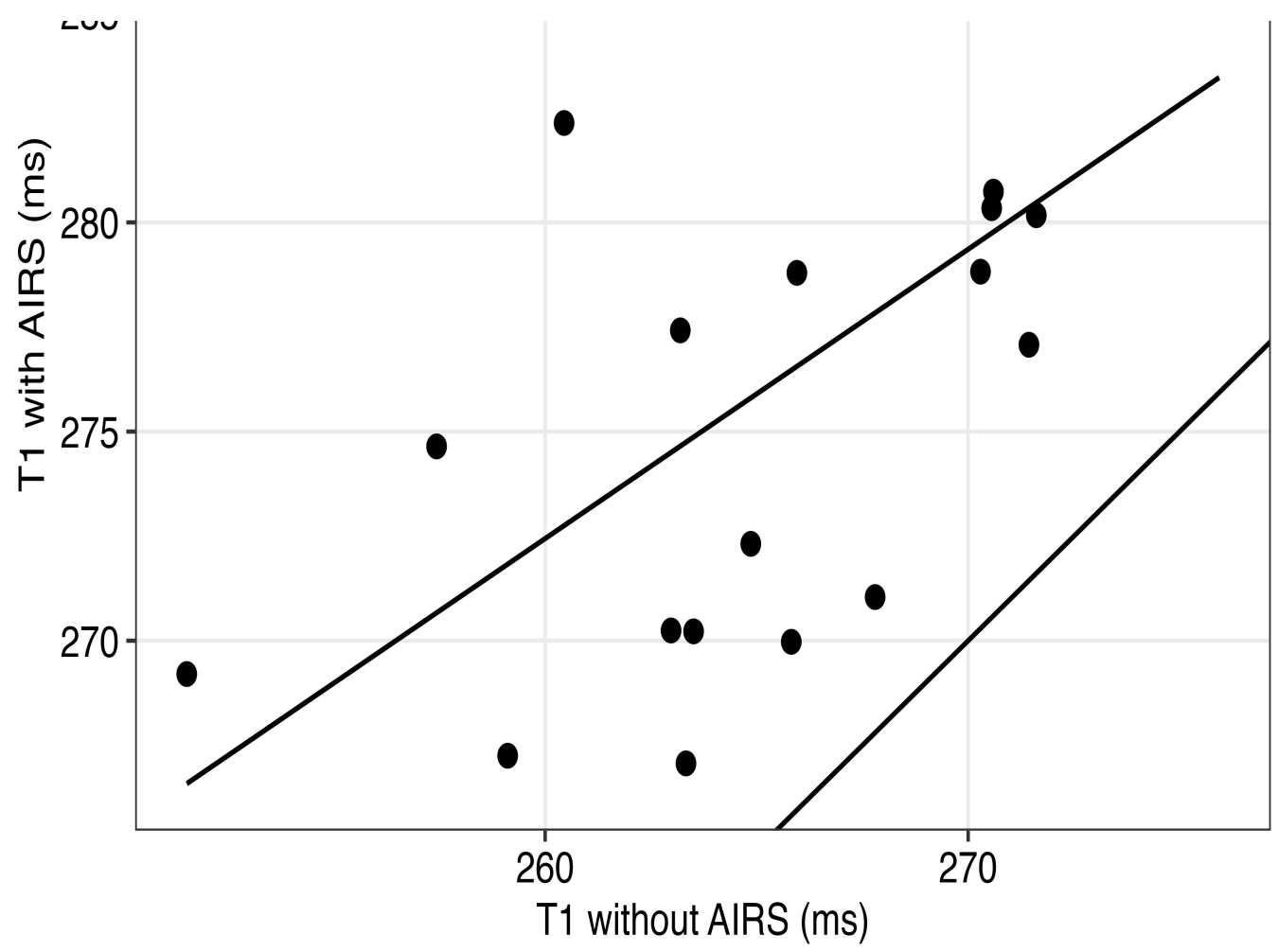

Scatterplot of T1 with AIRS (y-axis) vs without AIRS (x-axis) for 2ptIR. The identity line (y = x) and the OLS fit are shown. Each marker represents one animal × session pair. **Note**.—Regression coefficients and agreement summaries are provided in Table P5; raw paired values are listed in Table P6.

Abbreviations: 2ptIR = two-point inversion recovery; AIRS = AI-based reconstruction software; OLS = ordinary least squares.

Supplementary Figure P8. AIRS ON vs OFF (multiTR): identity line and OLS fit (normal rat liver; n = 20).

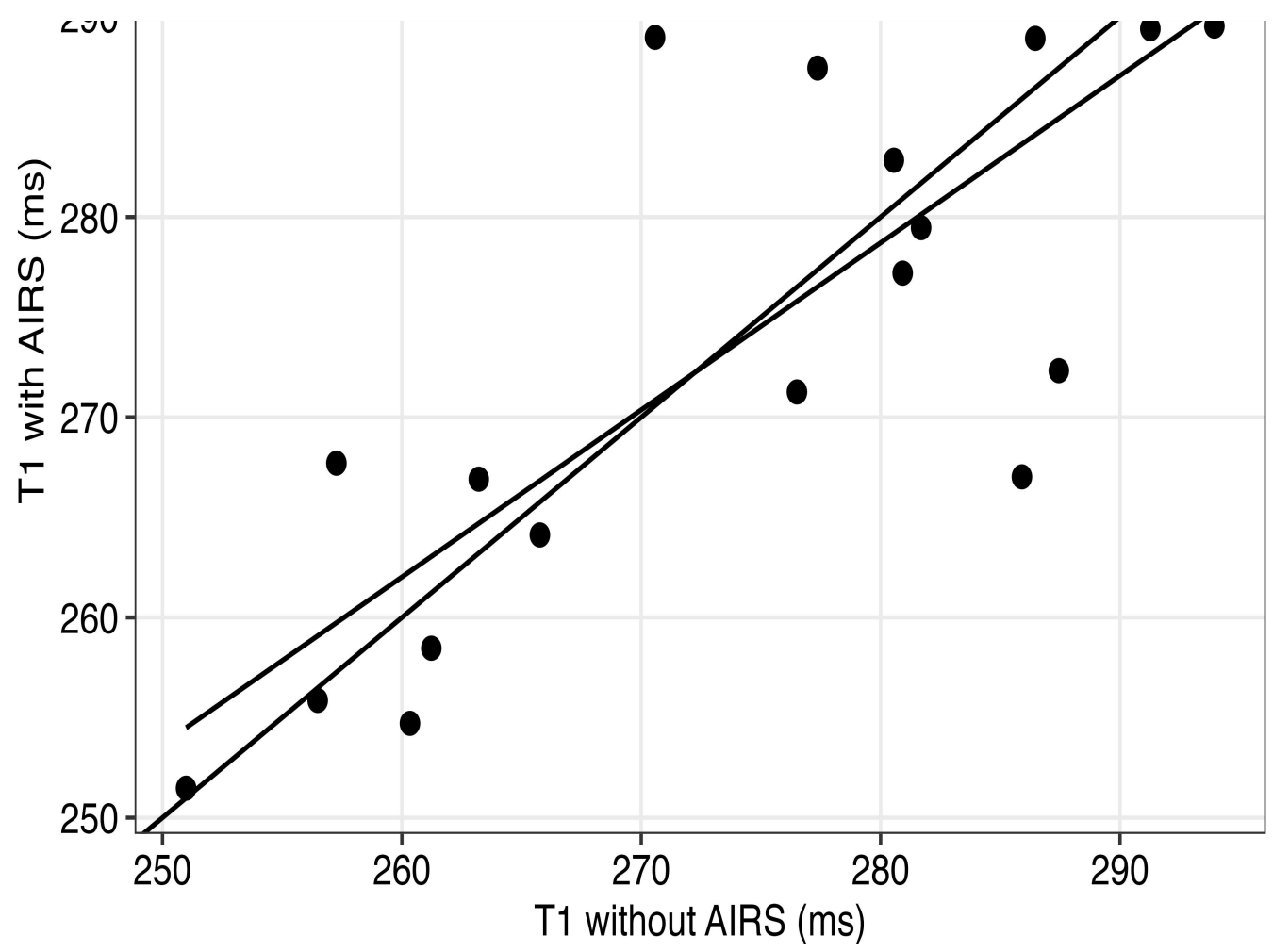

Scatterplot of AIRS ON (y-axis) vs AIRS OFF (x-axis) for multiTR. The identity line and the OLS fit are shown. **Note.**—Regression coefficients and agreement summaries are provided in Table P5; raw paired values are listed in Table P7.

Abbreviations: AIRS = AI-based reconstruction software; multiTR = multi-TR saturation recovery; OLS = ordinary least squares.

Supplementary Figure P9. Bland–Altman: test–retest (2ptIR, AIRS OFF; normal rat liver; n = 10).

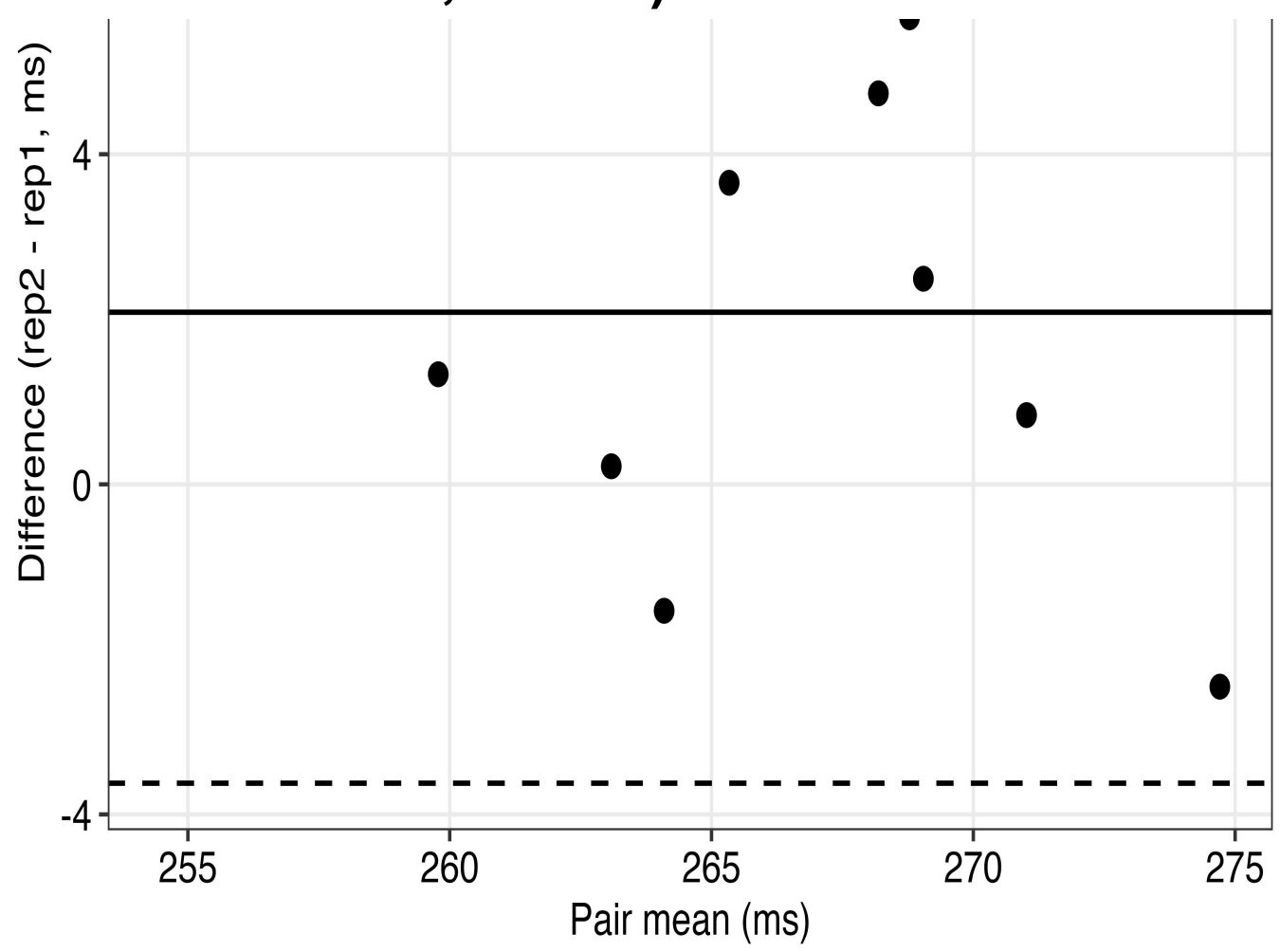

Bland–Altman plot for test–retest repeatability of 2ptIR (AIRS OFF). The difference is rep2 − rep1 (ms), and the mean is the pair mean. Solid line indicates bias; dashed lines indicate 95% LoA. **Note**.—Repeatability metrics (RC95, wSD, wCV, ICC, CCC) are provided in Table P4. **Abbreviations**: 2ptIR = two-point inversion recovery; AIRS = AI-based reconstruction software; CCC = concordance correlation coefficient; ICC = intraclass correlation coefficient; LoA = limits of agreement; RC95 = 95% repeatability coefficient; wCV = within-subject coefficient of variation; wSD = within-subject standard deviation.

Supplementary Tables (Appendix P)

Table P1. Phantom cross-calibration between 2ptIR and multiTR (n = 24 vial × session pairs).

Reconstruction	N	Slope	Intercept	R^2	RMSE (ms)	MAPE (%)	Bias (ms)	95% LoA (ms)
**AIRS OFF**	12	0.93	9.99	>.99	4.15	2.21	-3.59	-16.20 to 9.02
**AIRS ON**	12	0.97	-0.13	>.99	4.34	3.24	-5.34	-14.94 to 4.26

Note. — Agreement between the proposed rapid two-point inversion recovery (IR) method and the reference multi-TR saturation recovery method was evaluated using a T1 phantom (T1 range: ~100 to ~2000 ms), stratified by the use of deep-learning reconstruction (SwiftMR; AIRS Medical). "AIRS ON" represents the proposed protocol used for in vivo studies. Metrics are derived from ordinary least squares regression (Slope, Intercept, R^2) and Bland–Altman analysis (Bias, 95% limits of agreement). **Abbreviations:** AIRS = AI-based reconstruction software; LoA = limits of agreement; MAPE = mean absolute percentage error; RMSE = root mean square error; R^2 = coefficient of determination.

Table P2. Sensitivity to Flip Angle Miscalibration (T1 Phantom)

Reconstruction	N Vials	Mean % Change (FA +1°)
**AIRS OFF**	6	-2.31 ± 3.38
**AIRS ON**	6	-2.92 ± 3.46

Note.— Sensitivity of the two-point inversion recovery T1 mapping protocol to flip-angle miscalibration was evaluated by repeating the phantom acquisition with the flip angle increased by +1° relative to the nominal setting. Values are mean ± standard deviation of the relative percent change in T1 compared with the nominal flip angle acquisition, computed across six phantom vials. AIRS OFF indicates standard reconstruction, and AIRS ON indicates deep-learning reconstruction applied.

Abbreviations: AIRS = deep-learning–based image reconstruction (SwiftMR; AIRS Medical); FA = flip angle; T1 = longitudinal relaxation time.

Table P3. Phantom effect of AIRS reconstruction on T1 (paired AIRS ON vs OFF; n = 24).

Mapping Method	Mean Difference (ON - OFF)
**2ptIR**	-0.86 ± 0.66 ms
**multiTR**	-2.61 ± 4.47 ms

Note. — The mean difference (bias) introduced by deep-learning reconstruction (AIRS ON minus AIRS OFF) was evaluated across 6 phantom vials for both the proposed 2-point inversion recovery (2ptIR) and reference multi-TR methods.

Table P4. Repeatability and Agreement by Method and Reconstruction (Normal Rat Liver)

Method	Reconstruction	Bias (ms)	95% LoA (ms)	RC (ms)	wCV (%)	ICC	CCC
**2ptIR**	OFF	2.09	-3.62 to 7.79	5.71	0.77	0.84	0.83
**2ptIR**	ON	5.73	-9.55 to 21.00	15.28	1.99	0.15	0.14
**multiTR**	OFF	11.85	-21.33 to 45.04	33.19	4.34	0.10	0.09
**multiTR**	ON	12.10	-15.49 to 39.68	27.59	3.62	0.30	0.28

Note. — Test–retest repeatability metrics for in vivo normal rat liver T1 mapping (n=10 pairs per group). Bias indicates the mean difference between Session 2 and Session 1. Abbreviations: CCC = concordance correlation coefficient; ICC = intraclass correlation coefficient; LoA = limits of agreement; RC = repeatability coefficient; wCV = within-subject coefficient of variation.

Table P5. Agreement between Deep-Learning and Standard Reconstruction In Vivo (Normal Rat Liver)

Method	Slope (95% CI)	Intercept (95% CI)	R^2	Bias (ms)	95% LoA (ms)
**2ptIR**	0.69 (0.25, 1.13)	92.61 (-24.08, 209.30)	0.38	10.64	-0.31 to 21.58
**multiTR**	0.83 (0.55, 1.12)	44.99 (-32.76, 122.74)	0.68	-0.57	-16.71 to 15.56

Note. — Regression and Bland–Altman analysis comparing AIRS ON vs. AIRS OFF T1 values in normal rat livers (n=20 scans). Bias is defined as ON minus OFF.

Table P6. Paired T1 Measurements: 2ptIR Method (Normal Rat Liver)

Rat ID	Rep	T1 (AIRS ON)	T1 (AIRS OFF)
**R01**	1	269.97	265.82
**R01**	2	280.34	270.56
**R02**	1	286.48	275.93
**R02**	2	286.57	273.48
**R03**	1	280.74	270.60
**R03**	2	277.08	271.44
**R04**	1	270.22	263.51
**R04**	2	289.04	267.16
**R05**	1	272.32	264.86
**R05**	2	267.07	263.33
**R06**	1	269.20	251.53
**R06**	2	274.64	257.43
**R07**	1	267.25	259.11
**R07**	2	282.38	260.45
**R08**	1	270.24	262.98
**R08**	2	277.42	263.20

Rat ID	Rep	T1 (AIRS ON)	T1 (AIRS OFF)
**R09**	1	278.79	265.95
**R09**	2	280.17	271.61
**R10**	1	271.04	267.80
**R10**	2	278.82	270.29

Note. — Data are individual T1 relaxation times (in milliseconds) measured in the liver of 10 normal Wistar rats using the proposed rapid two-point inversion recovery (2ptIR) sequence. Each animal underwent two repeated acquisitions (Rep 1, Rep 2) in the same session to evaluate test–retest repeatability. Measurements were reconstructed with both the standard algorithm (AIRS OFF) and the deep-learning algorithm (AIRS ON) to assess the reconstruction effect.
Abbreviations: 2ptIR = two-point inversion recovery; AIRS = AI-based reconstruction software; T1 = longitudinal relaxation time.

Table P7. Paired AIRS OFF/ON values used for Fig. P8 (multiTR; normal rat liver).

Rat ID	Rep	T1 (AIRS ON)	T1 (AIRS OFF)
**R01**	1	297.22	292.70
**R01**	2	282.84	280.55
**R02**	1	272.33	287.44
**R02**	2	287.44	277.37
**R03**	1	255.86	256.48
**R03**	2	267.02	285.90
**R04**	1	266.91	263.21
**R04**	2	289.53	293.94
**R05**	1	277.20	280.92
**R05**	2	288.92	286.46
**R06**	1	251.47	250.98
**R06**	2	271.26	276.51
**R07**	1	279.47	281.69
**R07**	2	291.58	293.32
**R08**	1	264.12	265.77
**R08**	2	254.71	260.34

Rat ID	Rep	T1 (AIRS ON)	T1 (AIRS OFF)
**R09**	1	258.46	261.23
**R09**	2	289.40	291.27
**R10**	1	267.71	257.27
**R10**	2	288.97	270.58

Note. — Data are individual T1 relaxation times (in milliseconds) measured in the liver of 10 normal Wistar rats using the reference multi-TR saturation recovery sequence. The experimental design (n=10, 2 repetitions per animal) and reconstruction settings (AIRS ON/OFF) are identical to those in Table P6. These values serve as the in vivo reference standard for verifying the accuracy of the 2ptIR method. **Abbreviations:** AIRS = AI-based reconstruction software; multiTR = multi-TR saturation recovery; T1 = longitudinal relaxation time.
